# Large-scale transcriptomic and genomic analyses reveal a novel functional gene *SERPINB6* for chicken carcass traits

**DOI:** 10.1186/s40104-024-01026-3

**Published:** 2024-05-11

**Authors:** Di Zhao, Ranran Liu, Xiaodong Tan, Huimin Kang, Jie Wang, Zheng Ma, Haiquan Zhao, Hai Xiang, Zhengfen Zhang, Hua Li, Guiping Zhao

**Affiliations:** 1 Guangdong Provincial Key Laboratory of Animal Molecular Design and Precise Breeding, School of Life Science and Engineering, Foshan University, Foshan, China; 2 State Key Laboratory of Animal Biotech Breeding, Institute of Animal Sciences, Chinese Academy of Agricultural Sciences, Beijing, China; 3 Guangdong Tinoo’s Foods Group Co., Ltd., Qingyuan, China

**Keywords:** Carcass traits, Chicken, Genome, *SERPINB6*, Transcriptome

## Abstract

**Background:**

Carcass traits are crucial indicators of meat production efficiency. However, the molecular regulatory mechanisms associated with these traits remain unclear.

**Results:**

In this study, we conducted comprehensive transcriptomic and genomic analyses on 399 Tiannong partridge chickens to identify key genes and variants associated with carcass traits and to elucidate the underlying regulatory mechanisms. Based on association analyses with the elastic net (EN) model, we identified 12 candidate genes (*AMY1A*, *AP3B2*, *CEBPG*, *EEF2*, *EIF4EBP1*, *FGFR1*, *FOXD3*, *GOLM1*, *LOC107052698*, *PABPC1*, *SERPINB6* and *TBC1D16*) for 4 carcass-related traits, namely live weight, dressed weight, eviscerated weight, and breast muscle weight. *SERPINB6* was identified as the only overlapping gene by 3 analyses, EN model analysis, weighted gene co-expression network analysis and differential expression analysis. Cell-level experiments confirmed that *SERPINB6* promotes the proliferation of chicken DF1 cells and primary myoblasts. Further expression genome-wide association study and association analysis indicated that rs317934171 is the critical site that enhances *SERPINB6* expression. Furthermore, a dual-luciferase reporter assay proved that gga-miR-1615 targets the 3′UTR of *SERPINB6*.

**Conclusions:**

Collectively, our findings reveal that *SERPINB6* serves as a novel gene for chicken carcass traits by promoting fibroblast and myoblast proliferation. Additionally, the downstream variant rs317934171 regulates *SERPINB6* expression. These results identify a new target gene and molecular marker for the molecular mechanisms of chicken carcass traits.

**Supplementary Information:**

The online version contains supplementary material available at 10.1186/s40104-024-01026-3.

## Background

The worldwide attention given to food safety, particularly that of meat, eggs and milk, which are important sources of protein, has increased [1]. Meat is the most readily available source of various amino acids that the human body requires. In China, chicken is the second most widely consumed meat [2], and the demand for chicken meat is increasing annually. Local chicken breeds are preferred by native consumers over fast-growing chickens due to their delicate meat quality, including flavor and tenderness [3]. Additionally, carcass traits serve as important indicators of meat yield, which is a crucial economic trait of meat-type chickens. However, local chicken breeds have low meat production efficiency and their carcass trait regulatory mechanisms are largely unknown, making it difficult for them to meet the current demand for chicken meat.

Carcass traits are regulated by polygenic effects [4, 5]. Transcriptome sequencing (RNA-seq) revealed differential expression of *CRELD1* and *DNAJC30* in the breast muscle of white recessive rock chickens compared to Xinghua chickens [6]. RNA-seq analysis identified *PRKG2* as an important candidate gene associated with the carcass traits of Chinese Ningdu yellow chickens [7]. At present, transcriptomics is the most commonly used method for identifying candidate genes based on gene expression levels. However, at the genomic level, genome-wide association studies (GWAS) are frequently used to identify major genes or genetic markers [8–11]. For instance, a quantitative trait locus (QTL) region was identified on chromosome 4 of Beijing-You chickens. The region includes 7 significant single-nucleotide polymorphisms (SNPs) and 4 candidate genes (*LCORL*, *LAP3*, *LDB1* and *TAPT1*). These genes were found to be related to the dressed weight (DW) and eviscerated weight (EW) through a 60 K SNP chip and association analysis of 16 traits [12]. The results of a GWAS showed that the major-effect candidate gene *ADGRG6* was associated with the whole-thigh percentage and thigh percentage, while the major-effect candidate gene *DRD1* was associated with carcass traits such as carcass weight, EW, and thigh weight [13]. Numerous studies have demonstrated that combining GWAS and RNA-seq can aid in identifying candidate genes, mutations, or biological pathways [14] that affect complex traits. Tan et al. [15], found that the causal gene *SOX6* influenced the breast muscle yield of fast-growing broilers, based on genomic and transcriptomic evidence.

This study analyzed nearly 400 Tiannong partridge hens using RNA-Seq and whole-genome resequencing (WGS). Candidate genes were identified by combining large-scale transcriptomic data with multiple carcass traits, including live weight (LW), DW, EW, and breast muscle weight (BMW), through 3 analyses. Key loci affecting carcass traits were identified through expression genome-wide association study (eGWAS) and association analysis.

## Methods

### Ethics statement

The chickens were fed and handled in accordance with relevant national and international guidelines. The study and all experimental protocols were approved by the Laboratory Animal Welfare and Animal Experimental Ethical Inspection Board of Foshan University (No. 18091801).

### Animals and sample collection

All animals used in this study were provided by Guangdong Tinoo’s Foods Group Co., Ltd. All birds were raised on one of the company’s farms according to commercial feeding standards. After weighing, a total of 399 female Tiannong partridge chickens were slaughtered at 126 d using a mechanized slaughter line with moderate scalding water (61 °C). The left pectoral muscle was completely stripped and weighed, and samples of the same part of the right pectoral muscle were collected uniformly. The samples were quickly frozen in liquid nitrogen and stored at −80 °C for subsequent RNA and DNA isolation. We determined the DW, EW and BMW according to the performance terminology and measurements for poultry [16].

### RNA extraction and sequencing

Total RNA from breast muscle was isolated with TRIzol reagent (Invitrogen, Carlsbad, CA, USA). After quality control, the qualified RNA samples were subjected to reverse transcription and cDNA library construction as previously reported [17]. Briefly, RNA-seq was performed on an Illumina NovaSeq 6000 (Illumina, San Diego, CA, USA), and paired-end 150 bp reads were generated. FastQC v0.11.9 [18] was used to verify the quality of the raw sequence data for each sample. The trimmed transcriptome data was aligned to the chicken reference genome GRCg6a (GCA_000002315.5) using HISAT2 v2.1.0 [19]. Normalization of gene expression levels was carried out using DESeq2 v1.24.0 [20].

### Elastic net-1 standard error model for association analysis

Four statistical models, namely linear mixed model (LMM), least absolute shrinkage and selection operator (LASSO), ridge regression (RR), and elastic net (EN), were used to analyze the associations between gene expression levels and carcass traits of 381 chickens. 18 individuals with phenotypic deletions were eliminated prior to analysis [21]. According to our previous research, the simulation study results showed that the elastic net-1 standard error (EN-1SE) model, which does not involve significance testing, was the best method for analyzing the associations between expression levels and BMW. This was true in terms of both the type I error rate and the detection power [21]. Therefore, we used the EN-1SE model [22] to analyze the association between gene expression and LW, DW, and EW. Briefly, candidate genes for 4 traits were detected using EN with tuning parameters determined based on the one standard error rule (based on the results of the simulation study). All data, including phenotypic records and gene expression levels, were scaled to a mean of zero and one unit of variance prior to association analyses for both the simulation and the empirical studies. The EN models are multi-locus models in which the expression levels of all genes are analyzed together, and EN is classified as regularized linear regression model, which is the basic linear regression model:

$$y=\varvec{\mu}1+\sum_{i=1}^mX_i\varvec{b}\varvec{_i}+\varvec{e}$$

where ***µ*** is the population mean, 1 is a vector of 1s, the independent variable *X*_*i*_ is a vector of expression values of the *i*^th^ gene, and ***b***_***i***_ is the effect of the *i*^th^ gene on the trait under study.

The EN model uses a mixture of *l*_*1*_ and *l*_*2*_ penalties and can be formulated as follows:

$$\widehat{\varvec{b}}\left(EN\right)={argmin}_{b}\left\{{\left(y-\varvec{\mu}1-X\varvec{b}\right)}^{{\prime }}\left(y-\varvec{\mu}1-X\varvec{b}\right)+\lambda \sum _{i=1}^{m}\left[\left(1-\alpha \right){\varvec{b}}_{\varvec{i}}^{2}+\alpha \left|{\varvec{b}}_{\varvec{i}}\right|\right]\right\}$$

where the value of the second parameter, *α*, is also determined via cross-validation. The Wald test was used to test whether the estimates of ***b***_***i***_ from the 4 models were significantly different from zero. Under the null hypothesis that ***b***_***i***_ = 0, the Wald test statistic is:

$${\textit{W}}_\textit{i}\textit{=}\frac{\textit{b}_\textit{i}^{\mathit2}}{{\textit{var(b}}_\textit{i}\textit{)}}$$

and follows approximately a one-degree of freedom Chi-square distribution. The GLMNET/R package was used to calculate EN-1SE [23]. In addition, EN automatically performs variable selection. We simply selected non-zero effect genes as candidate genes in the analyses using EN-1SE model.

### Weighted gene co-expression network analysis (WGCNA)

A weighted gene co-expression network was constructed in the R environment using the standard pipeline, based on gene expression levels. A total of 350 individuals were used for WGCNA [24], and outlier samples were removed based on the criterion mean ± 3 standard deviations. Briefly, the soft threshold (*β* = 6) was determined according to the scale-free distribution (*R*^2^ > 0.85). To determine if 2 genes have similar expression patterns, a soft threshold is typically set for screening. Genes with expression levels above the soft threshold are considered similar. The soft threshold is not a fixed value, but rather an idempotent that reflects the importance of a numerical value based on its magnitude. WGCNA uses correlation coefficient weighting, which involves raising the gene correlation coefficient to the N^th^ power to ensure that the connections between genes in the network follow a scale-free distribution. The gene network was constructed using step-by-step and dynamic cutting methods to detect modules (minModuleSize = 50, mergeCutHeight = 0.25). The module with the highest correlation coefficient was identified as the target module. The module membership (MM) and gene significance (GS) were calculated in the target module [25]. Within the module, the hub genes were defined according to the criteria GS > 0.2 and MM > 0.6.

### Differential expression analysis and pathway enrichment

To further screen for differentially expressed genes (DEGs) related to the 4 carcass traits, we separately sorted 350 individuals based on high to low values for the LW, DW, EW and BMW, and a total of 60 birds were divided into the high group (*n* = 30) and the low group (*n* = 30) (Additional file 1: Fig. S1). The genes with a fold change > 1.5 and *P* < 0.05 were identified as DEGs. Similarly, DEGs from the chicken embryonic fibroblast line (DF1 cells), were screened using the criteria (fold change > 1.5 and *P* < 0.05). Simultaneously, all of these DEGs were subjected to perform Kyoto Encyclopedia of Genes and Genomes (KEGG) and Gene Ontology (GO) enrichment analyses using KOBAS 3.0 (http://kobas.cbi.pku.edu.cn). A *P* value of 0.05 was used as the threshold for significant pathway enrichment.

### Cell culture

DF1 cells (from the cell bank of the Chinese Academy of Sciences) and human embryonic kidney 293 cells (HEK293 cells, from ATCC) were cultured in high-glucose DMEM medium (Gibco, CA, USA) supplemented with 10% fetal bovine serum (FBS, Gibco, New Zealand) and 1% penicillin‒streptomycin (Gibco, CA, USA) under 5% CO_2_ at 37 °C. In addition, the chicken primary myoblasts (CPMs) were isolated from the breast muscle tissue of healthy less than 10-day-old commercial chickens in a sterile environment. Briefly, the breast muscle tissue was washed no less than 3 times with PBS, and the breast muscle tissue was cut to the size of rice grains and digested with collagenase. At the same time, erythrocyte lysates were used to remove erythrocytes. The isolated CPMs were cultured in DMEM/F12 medium (Gibco, CA, USA) supplemented with 10% FBS and 1% penicillin–streptomycin. CPMs were used in subsequent experiments within 5 generations.

### RNA oligonucleotides, plasmid construction and cell transfection

*SERPINB6* small interfering RNAs (siRNAs) were provided by RiboBio Co., Ltd. (Guangzhou, China). The miR-1615 mimic, mimic negative control (mimic NC), miR-1615 inhibitor and inhibitor negative control (inhibitor NC) were synthesized by GenePharma Co., Ltd. (Shanghai, China), based on oligonucleotide sequences (Additional file 2: Table S1).

For the construction *SERPINB6* overexpression plasmid, the full-length sequence of *SERPINB6* was amplified from chicken breast muscle cDNA by PCR and cloned and inserted into the empty expression plasmid pcDNA3.1 using the NheI and XhoI restriction sites. For the pGL4.18 dual-luciferase reporter vector, the segment sequence of the 3′UTR of *SERPINB6* containing the putative gga-miR-1615 binding sequence was amplified by PCR and then subcloned and inserted between the XhoI and HindIII restriction sites in the pGL4.18 dual-luciferase reporter vector. The *SERPINB6* 3′UTR mutant plasmid was generated by changing the binding site of gga-miR-1615 from AGCTGCCA to AGCTACCA. PCR amplification was performed for mutagenesis and DpnI digestion to remove the parental DNA.

Transfection was performed when the cells were at high confluence (> 80%), and in 96-well plates when the cell confluence was approximately 70%. Transient transfection of plasmids and oligonucleotides was performed using Lipofectamine^™^ 3000 reagent (Invitrogen, CA, USA). Liposomes, plasmids, and oligonucleotides were diluted with modified minimal essential medium (Opti-MEM, Gibco, CA, USA). The diluted liposomes were mixed with diluted plasmids or oligonucleotides, and then incubated at room temperature for 15 min to prepare the transfection solution. For RNA oligonucleotides, a concentration of 150 nmol/L was used. The transfection solution was added to the cell medium and incubated for 6–8 h to complete the transfection, after which the cell medium was changed. TransIT-TKO transfection reagent (Mirus Bio, Germany) was used only for the transfection of siRNA into CPMs. The qPCR was performed at 24 h after cell transfection.

### Gene expression analysis of *SERPINB6*

Total cellular RNA was extracted using TRIzol according to the manufacturer’s instructions, and the concentration and integrity of the RNA was measured using an Agilent 2100 Nano and by gel electrophoresis. The RNA obtained from *SERPINB6* overexpressing DF1 cells was subjected to RNA-seq according to the methods described above. cDNA was generated from 2 μg of RNA using FastKing gDNA Dispelling RT SuperMix (TIANGEN Biotech Co., Ltd., Beijing, China). The obtained reverse transcription products were subsequently mixed with PowerUp™ SYBR™ Green Master Mix (Applied Biosystems, Waltham, MA, USA), primers, and double-distilled water (ddH_2_O) to produce a 10 μL RT‒PCR system. Primers were designed using the National Center for Biotechnology Information (NCBI) database based on the coding sequence of *SERPINB6* (F: 5′–CCTCAGAACTGCTAACCGACTT–3′, R: 5′–CTTCTCTTCCACCCAGCCATT–3′) and flanking genes (Additional file 3: Table S2) in the 5′ to 3′ direction. Three technical replicates were performed for each sample. The RT–PCR conditions were as follows: 94 °C for 3 min, followed by 40 cycles of 94 °C for 3 s and an annealing temperature for 34 s. *GAPDH* and *RPL32* were selected as the internal controls (Additional file 3: Table S2). According to the RT–PCR results, the expression level was calculated using the 2^−ΔΔCT^ method [26]. Three independent biological replicates were used for each experimental group.

### CCK8 and 5-ethynyl-2-deoxyuridine (EdU) proliferation assays

Cell proliferation was measured using Cell Counting Kit-8 (CCK8) and EdU assays. DF1 cells and CPMs were seeded in 96-well plates for 24 h, 10 μL of CCK8 reagent was added to each well at 12, 24, 36, and 48 h post-transfection, and the plates were then incubated for 2 h. The absorbance value of each sample was measured using a microplate reader (Thermo, Waltham, MA, USA) at 450 nm. EdU assays were performed using the Cell-Light^TM^ EdU Apollo 488 In Vitro kit (RiboBio Co., Ltd., Guangzhou, China) according to the manufacturer’s instructions. Briefly, at 24 h after transfection, the cells were exposed to 50 mmol/L EdU for 2 h. The cells were fixed with 4% paraformaldehyde and permeabilized with 0.5% Triton X-100. Subsequently, the cells were incubated in Apollo reaction solution for 1 h and stained with Hoechst 33342 for 0.5 h. The cells were further analyzed by calculating the ratio of EdU-positive cells to the total number of cells. Three independent biological replicates were used for each experimental group. Three fields were randomly selected from each well, and the images were captured using a DMI3000B microscope (Leica, Wetzlar, Germany).

### Whole-genome sequencing

Genomic DNA from each individual was extracted from breast muscle samples using the phenol–chloroform method. The DNA quality was assessed by agarose gel electrophoresis. A DNA library (paired-end, 2 × 150 bp) was constructed for each DNA sample, and all libraries were sequenced on the Illumina Nova 6000 sequencing platform. Overall, approximately 2.4 Tb raw data was generated. Quality control criteria for the reference panel included an individual call rate ≥ 90%, SNP call rate ≥ 90%, and minor allele frequency (MAF) ≥ 0.05, as implemented in PLINK v1.9 [27]. We obtained 11,766,230 filtered SNPs on chromosomes 1–33, Z and W in 357 chickens.

### Variant calling, quality control, and annotation

Raw reads were first trimmed using Trimmomatic v0.36 with default parameters [28], and only paired quality reads were retained for the subsequent analysis. The filtered reads for all individuals were aligned to the chicken reference genome (GRCg6a) using Burrows‒Wheeler Aligner (BWA) v0.7.17 [29]. The resulting Sequence Alignment Map (SAM) files were converted to Binary Alignment Map (BAM) format and then sorted by coordinates and indexed using SAMtools v1.12 [30]. Polymerase chain reaction (PCR) duplicates were removed using PICARD v2.26. SNPs were first called using the HaplotypeCaller function in Genome Analysis Toolkit (GATK) v4.2.2 [31], and the genomic variant call format (gVCF) file was acquired for each chicken was acquired. The functions CombineGVCFs and GenotypeGVCFs were used to combine all the gVCF files and to jointly call SNPs together. In addition, SNPs were removed using GATK with specific standards: Quality Score < 30.0, QualByDepth < 2.0, FisherStrand > 60.0, RMSMappingQuality < 40.0, StrandOddsRatio > 3.0, MappingQualityRankSumTest < −12.5, and ReadPosRankSum < −8.

### Expression GWAS and association analysis

Association analysis of *SERPINB6* expression was performed using the mixed linear model in Genome-wide Efficient Mixed Model Association (GEMMA) v0.98.4 [32]. An eGWAS of *SERPINB6* expression was performed according to the following formula:

$$y=\alpha +X{\varvec{\beta}}+{\varvec{u}}+{\varvec{\varepsilon}}$$

where *y* is the vector of phenotypic values, *α* is the corresponding coefficient including the intercept, *X* is the genotype vector, ***β*** is the SNP effect vector, ***u*** is the random effect, and ***ε*** is the residual. Briefly, a Bonferroni correction was applied to adjust for multiple testing. The genome-wide significance threshold was 0.05/11,766,230, and the genome-wide potential significance threshold was 1/11,766,230. Visualization of the eGWAS results was performed using the qqman package of the R software.

We extracted all SNPs of *SERPINB6*, including those in the promoter region spanning 2 kb upstream of the gene. Based on the general linear model (GLM), correlation analyses of 4 traits and all SNPs of *SERPINB6* were performed using PLINK v1.9 [27]. No fixed effects (e.g., sex, batch) were included in this model. Significant SNPs affecting each trait were screened using *P* < 0.05 as a threshold.

### Target mRNA prediction

To further explore the underlying molecular mechanisms by which *SERPINB6* regulates carcass traits, its upstream and downstream targets were predicted using bioinformatics software, including PROMO software (http://alggen.lsi.upc.es/) and miRDB software (http://www.mirdb.org/mirdb/index.html).

### Luciferase reporter assays

HEK293 cells were seeded in 24-well plates. After co-transfection with the pGL4.18-WT-SERPINB6-3′UTR (wild type, WT) or pGL4.18-MT-SERPINB6-3′UTR (mutant type, MT) plasmid and the gga-miR-1615 mimic or mimic NC, firefly and Renilla luciferase activities were measured at 36 h post-transfection using a Dual-GLO Luciferase Assay System Kit (Promega, Madison, Wisconsin, WI, USA) according to the manufacturer’s instructions. Luminescence was measured using a Fluorescence/Multi-Detection Microplate Reader (BioTek, Vinusky, VT, USA), and firefly luciferase activity was normalized to Renilla luminescence in each well. Each sample included 3 technical replicates, and 3 independent biological replicates were performed.

### Statistical analysis

Statistical analyses were performed using SPSS 26.0 software or a custom script in the R environment. The Kruskal‒Wallis test was used to determine the differences in SNPs between the 3 genotypes. The GLM was used to compare the differences in carcass trait phenotypes and *SERPINB6* expression, with *P* < 0.05 was set as the significance threshold. Spearman correlation analysis was used to estimate the relationships between 4 carcass traits (LW, DW, EW, and BMW). All the data are presented as the mean ± 3 standard deviations from 3 biological replicates.

## Results

### The 4 carcass traits are highly correlated

Descriptive statistics of the carcass traits of 381 Tiannong partridge chickens with phenotypes are shown in Table 1. At 126 d of age, the LW, DW, EW and BMW were 1,513.41 g (CV = 11.98%), 1,343.99 g (CV = 12.19%), 1,014.29 g (CV = 12.41%) and 167.36 g (CV = 13.92%), respectively. In addition, the 4 carcass traits showed a highly significant correlation, such as the strong correlation between the EW and BMW (*r* = 0.74, *P* < 0.01), and the close relationships between the EW and LW and between the EW and DW are also reflected in the heatmap (Additional file 4: Fig. S2).

**Table 1:** Descriptive statistical analysis of 4 carcass traits

Trait, g	Mean	SD^1^	Max	Min	CV, %^2^
Live weight	1,513.41	181.36	2,200.00	1,070.00	11.98
Dressed weight	1,343.99	163.77	2,000.00	944.50	12.19
Eviscerated weight	1,024.29	127.09	1,562.90	751.50	12.41
Breast muscle weight	167.36	23.29	286.00	110.80	13.92

^1^*SD* Standard deviation

^2^*CV* Coefficient of variation

### The 12 candidate genes were identified using EN-1SE model

**Fig. 1:**
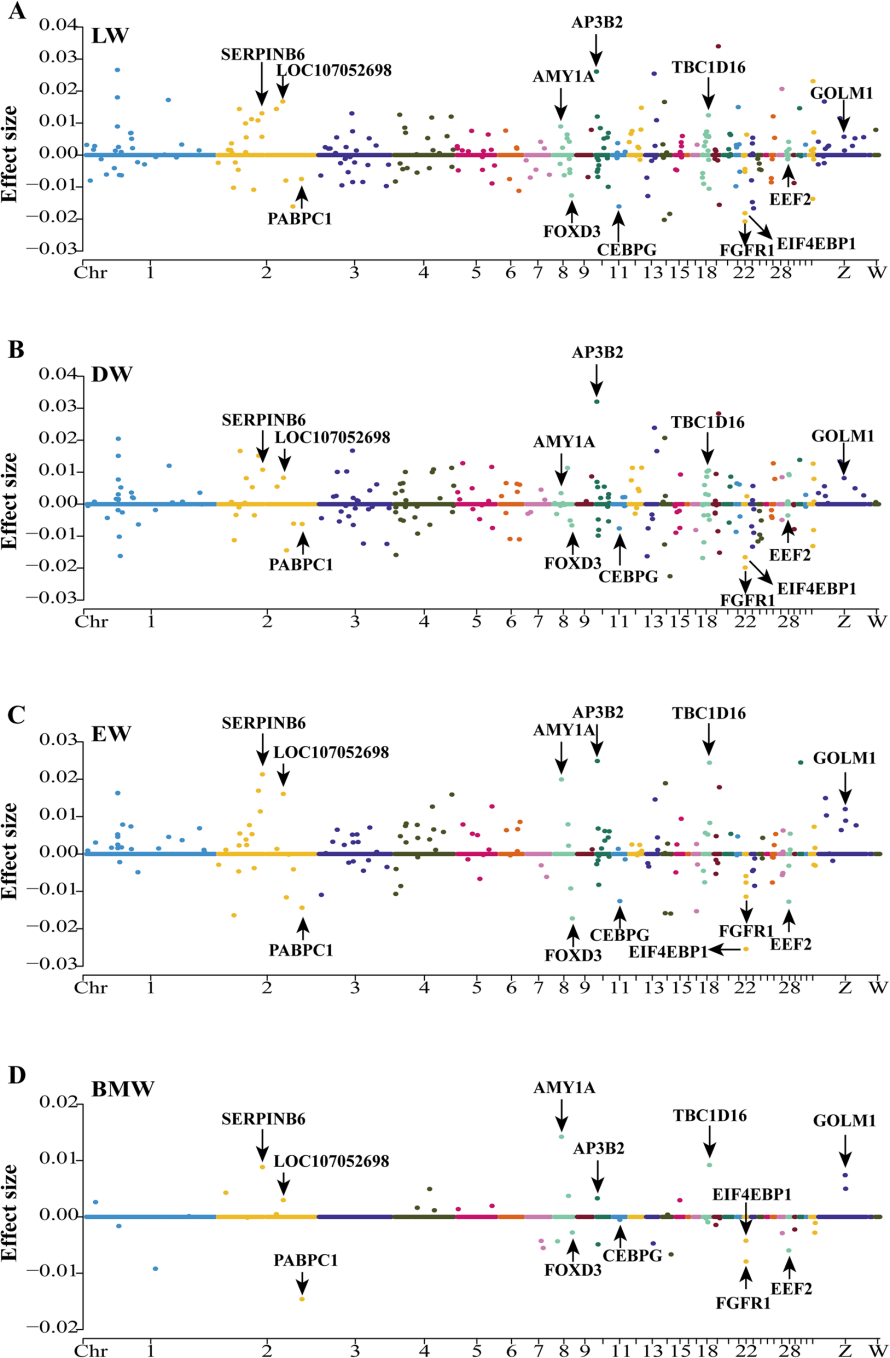
Identification of candidate genes for (**A**) live weight (LW), **B** dressed weight (DW), **C** eviscerated weight (EW) and **D** breast muscle weight (BMW) based on association analyses using EN-1SE. The estimated effect sizes and Manhattan plot of the genes are shown. The parameters of EN-1SE were determined based on the one standard error rule. Each dot represents a gene

In this study, a total of 15,092 coding genes (excluding non-coding genes) were selected from 399 chickens for subsequent analysis. To identify candidate genes for the traits analyzed, we estimated effect sizes for each trait. The estimated effect sizes of genes between traits and expression levels are shown based on the EN-1SE model for phenotypic associations in Fig. 1. We identified 400 genes in total that had non-zero effects on 4 carcass traits using the EN-1SE model, among which 12 genes had non-zero effects on the LW, DW, EW and BMW (Additional file 5: Table S3), i.e., *AMY1A*, *AP3B2*, *CEBPG*, *EEF2*, *EIF4EBP1*, *FGFR1*, *FOXD3*, *GOLM1*, *LOC107052698*, *PABPC1*, *SERPINB6* and *TBC1D16*. Therefore, these 12 genes were identified as candidate genes based on the EN-1SE model (Additional file 6: Fig. S3 and Table 2). Additionally, we found that *SERPINB6* and *TBC1D16* had large effects and were among the top 5 genes affecting the EW and BMW (Fig. 1C and D).

**Table 2:** Screening candidate genes based on the EN-1SE model and WGCNA

	**Genes**	**Screening criteria**
EN-1SE^1^	*AMY1A*, *AP3B2*, *CEBPG*, *EEF2*, *EIF4EBP1*, *FGFR1*, *FOXD3*, *GOLM1*, *LOC107052698*, *PABPC1*, *SERPINB6*, *TBC1D16*	Effect size > 0
WGCNA^2^	*ASH2L*, *BTBD2*, *CREBBP*, *CSNK2A2*, *DNAJA3*, *EDC3*, *EI24*, *EPFIP1L*, *HNRNPA1*, *INTS4*, *LCMT1*, *LMF2*, *LOC101748546*, *LOC101749377*, *LOC107057564*, *NSD1*, *PTP4A3*, *RNPS1*, *SERPINB6*, *SF3B4, SMG5*, *SYDE2*, *TADA3*, *UBE2O*, *YTHDF2*, *ZNF335*	Gene significance > 0.2, Module membership > 0.6

^1^*EN-1SE* Elastic net-1 standard error

^2^*WGCNA* Weighted gene co-expression network analysis

### Hub genes were screened by WGCNA

**Fig. 2:**
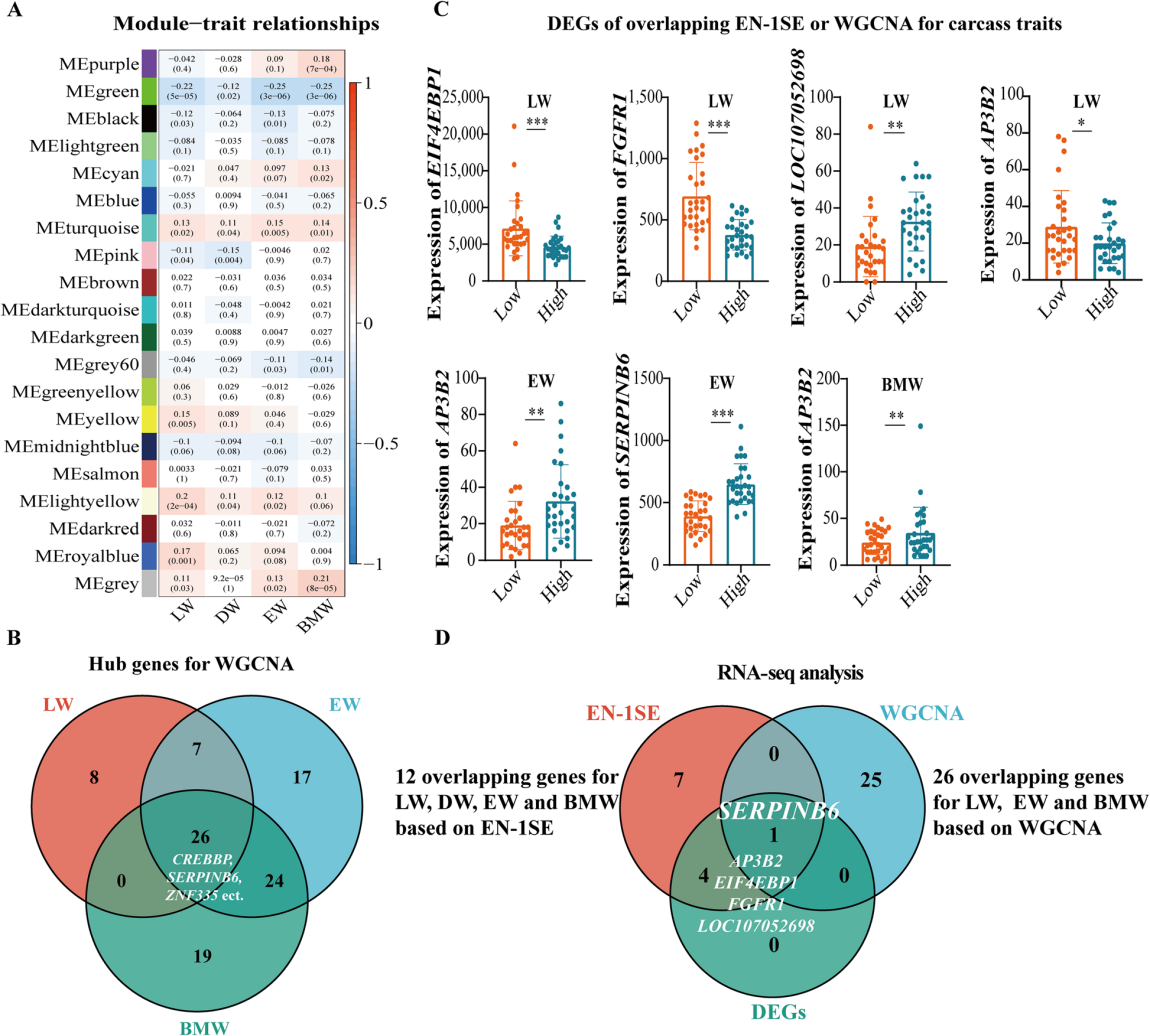
A candidate functional gene, *SERPINB6*, was identified by 3 analyses, including EN-1SE model analysis, WGCNA and differential expression analysis. **A** Correlation analysis between the module and carcass traits (*n* = 350). Red represents a positive correlation, blue represents a negative correlation, and a darker color indicates a stronger the correlation. **B** Venn diagram of 26 overlapping hub genes associated with carcass traits based on WGCNA. **C** Histogram of differential expression of overlapping genes identified from the differential expression analysis, EN-1SE model or WGCNA. Low indicates the low group, and High indicates the high group. *: *P* < 0.05, **: *P* < 0.01, ***: *P* < 0.001. **D** Venn diagram of 1 overlapping gene (*SERPINB6*) associated with the EW based on the genes with non-zero effects, hub genes and DEGs. EN-1SE: elastic net with parameters determined based on the one standard error rule, WGCNA: weighted gene co-expression network analysis, DEGs: differentially expressed genes, LW: live weight, DW: dressed weight, EW: eviscerated weight, BMW: breast muscle weight

To determine the biological relationships and possible functions of the hub genes, we constructed networks using WGCNA. First, we set the height to 1,200,000 based on the basis of the gene expression level and eliminated 6 outlier samples (Additional file 7: Fig. S4A). The optimal soft threshold was determined (*β* = 6) by taking *R*^*2*^ > 0.85 as the standard, and 24 modules were obtained (Additional file 7: Fig. S4B). Then, the modules with a similarity greater than 0.7 were clustered according to the module eigengenes (Additional file 7: Fig. S4C). Finally, 20 modules were obtained (Additional file 7: Fig. S4D). The correlation coefficients and *P* values between the modules and the 4 carcass traits were obtained. We found that the target module was the green module, which was considered to be the most clearly relevant module. The green module had the highest correlation with the LW, EW and BMW (*r* = −0.22, *P* = 5.0 × 10^–5^; *r* = −0.25, *P* = 3.0 × 10^–6^; *r* = −0.25, *P* = 3.0 × 10^–6^) (Fig. 2A). A total of 101 hub genes of the target modules were determined using the criteria GS > 0.2 and MM > 0.6 (Additional file 8: Table S4). Twenty-six hub genes were screened for the LW, EW and BMW, including *CREBBP*, *SERPINB6* and *ZNF335* (Fig. 2B and Table 2).

### *SERPINB6* is a candidate gene for carcass traits

Furthermore, we screened hundreds of DEGs in the high and low groups for each phenotype (Additional file 9: Fig. S5, Additional file 10: Table S5). Only 5 of the 12 candidate genes (*AP3B2*, *EIF4EBP1*, *FGFR1*, *LOC107052698* and *SERPINB*6) based on the EN-1SE model were DEGs that were differentially expressed in at least one phenotype (Fig. 2C). The expression of *SERPINB6* was higher in the high EW group than in the low EW group. Among the 3 analyses mentioned above, *SERPINB6* was the only overlapping gene for the EW (Fig. 2D). Therefore, *SERPINB6* was confirmed as the key candidate gene for carcass traits.

### *SERPINB6* promotes cell proliferation

**Fig. 3:**
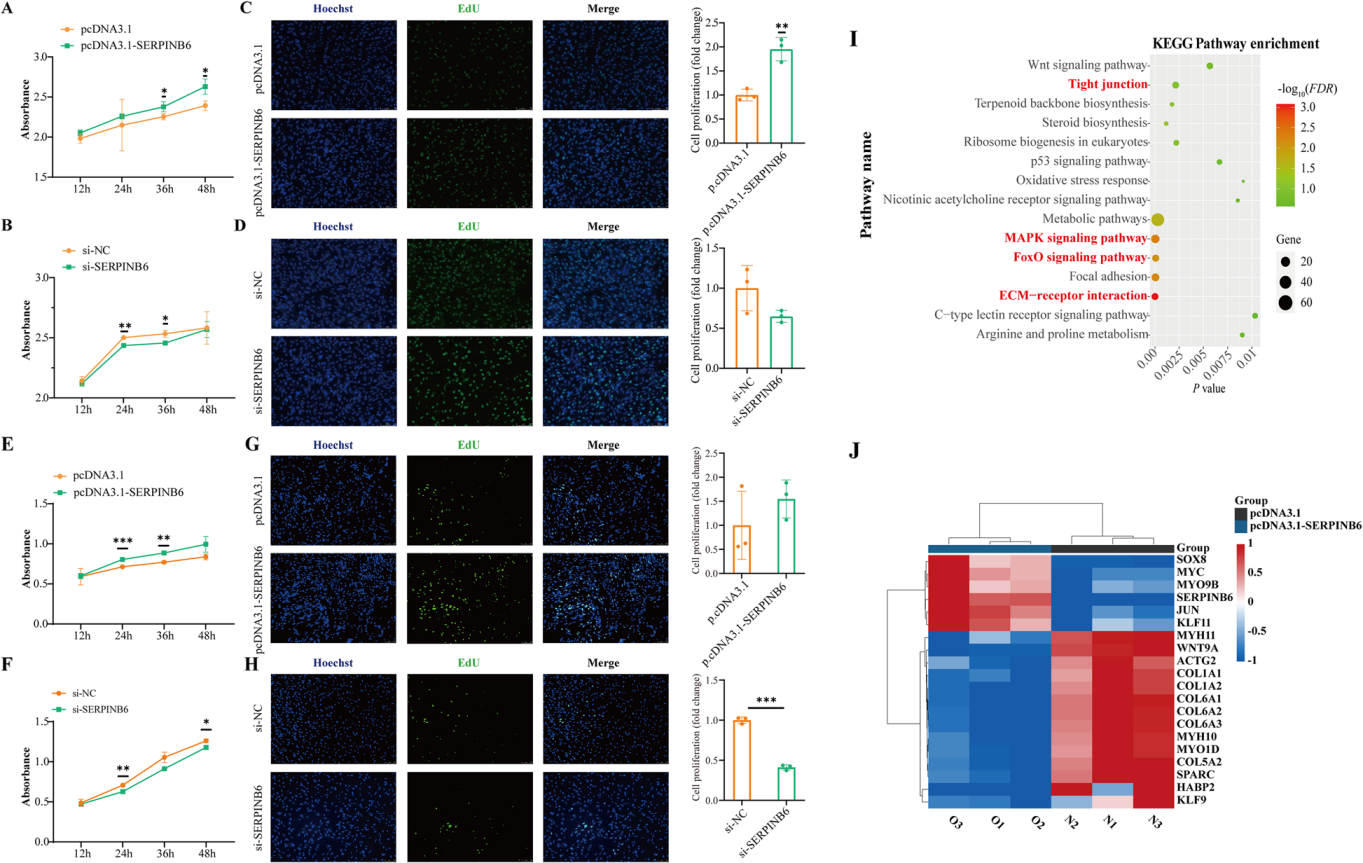
Verification the function of *SERPINB6* at the cellular level. **A** and **B** Cell growth after the transfection of DF1 cells with the empty vector pcDNA3.1 and pcDNA3.1-SERPINB6 or si-NC and si-SERPINB6. *: *P* < 0.05, **: *P* < 0.01. **C** and **D** The proliferation of transfected DF1 cells assessed by EdU incorporation. **: *P* < 0.01, ***: *P* < 0.001. **E** and **F** Cell growth following the transfection of CPMs with the empty vector pcDNA3.1 and pcDNA3.1-SERPINB6 or si-NC and si-SERPINB6. *: *P* < 0.05, **: *P* < 0.01, ***: *P* < 0.001. **G** and **H** Proliferation of transfected CPMs was assessed by EdU incorporation. **: *P* < 0.01. **I** The top 15 KEGG pathways enriched in DEGs in *SERPINB6*-overexpressing cells. **J** Heatmap of DEGs. N indicates pcDNA3.1, and O indicates pcDNA3.1-SERPINB6. DEGs: differentially expressed genes

To determine whether exogenous expression of *SERPINB6* could promote cell proliferation, we performed CCK8 and EdU assays. First, a *SERPINB6* was successfully overexpressed using a SERPINB6 overexpression vector (Additional file 11: Fig. S6A), and SERPINB6-si-3 (si-SERPINB6) had the highest interference efficiency among the 3 siRNAs in DF1 cells (Additional file 11: Fig. S6B). *SERPINB6* overexpression significantly improved the proliferation of DF1 cells (Fig. 3A), while a lower absorbance value was observed after si-SERPINB6 transfection compared to the control in the CCK8 assay (Fig. 3B). *SERPINB6* overexpression resulted in increased EdU positivity compared to the control, but *SERPINB6* knockdown significantly decreased EdU incorporation (Fig. 3C and D). Therefore, *SERPINB6* promotes the proliferation of DF1 cells. Moreover, we used CPMs as a cellular model for further validation, and the results were consistent with those obtained for DF1 cells (Fig. 3E–H and Additional file 11: Fig. S6C–D).

After overexpressing *SERPINB6* at the cellular level, we investigated the role of this gene in this pathway. We found 1,576 DEGs (1,023 upregulated and 553 downregulated) (Additional file 11: Fig. S6E). Further enrichment analyses revealed that 38 KEGG pathways and 456 GO terms were enriched (Additional file 12: Table S6). Moreover, all of these enriched pathways, which included KEGG pathways (tight junction, ECM-receptor interaction, MAPK signaling and focal adhesion) and GO terms (extracellular space, cytoplasm and cytosol) (Fig. 3I and Additional file 11: Fig. S6F), were involved in the proliferative function of *SERPINB6*. Among them, 20 major DEGs were enriched in the above-mentioned pathways and promoted or inhibited cell proliferation at the transcriptional level (Fig. 3J). The 20 DEGs revealed potential interactions between ACTA2, COL6A1 and SERPINB6 at the protein level (Additional file 13: Fig. S7).

### Three loci were screened by eGWAS and association analysis

**Fig. 4:**
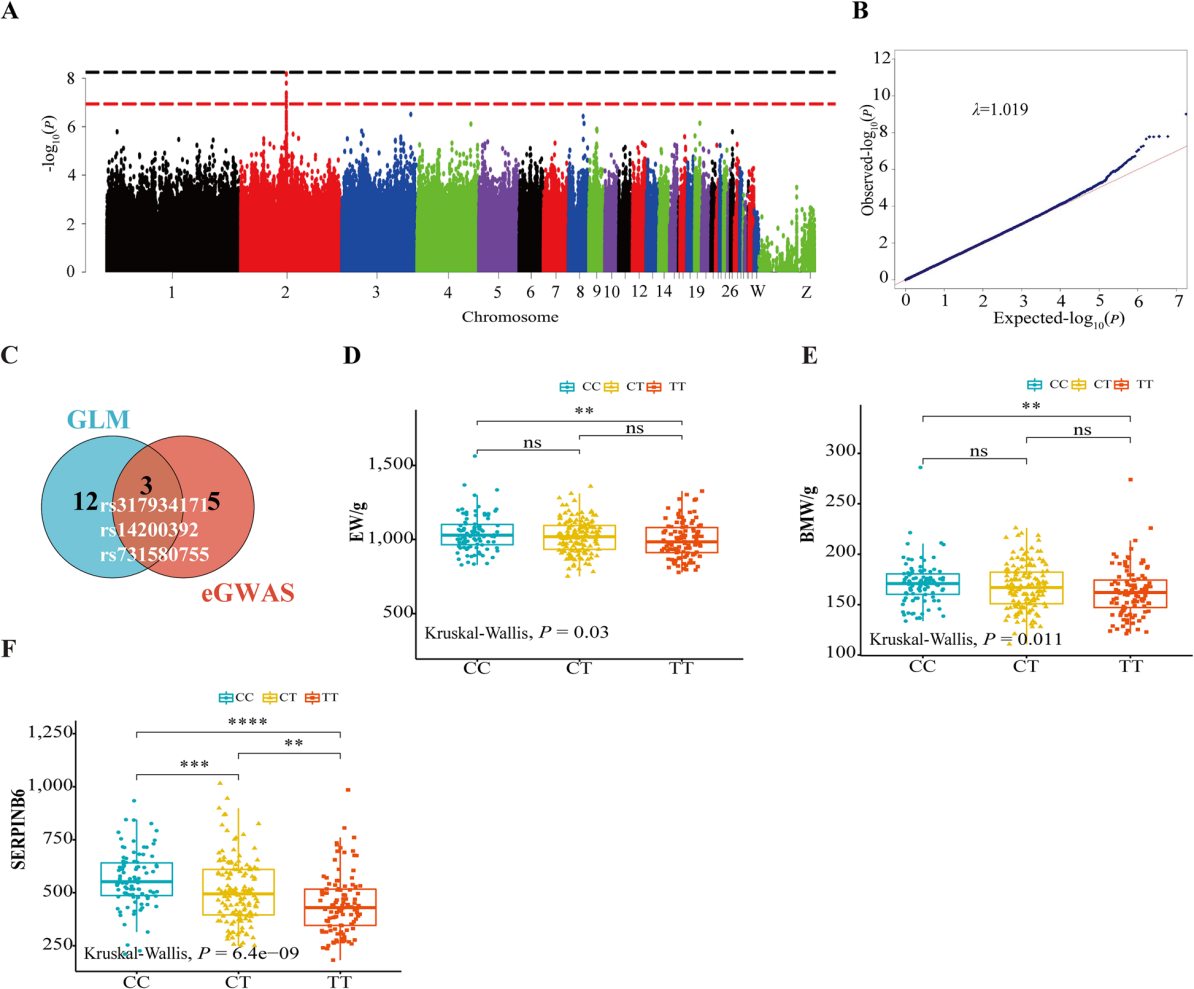
eGWAS of *SERPINB6* expression and effects of different genotypes of rs317934171. **A** and **B** Manhattan plots and quantile‒quantile (Q‒Q) plots of eQTLs for *SERPINB6*. Each dot represents a SNP. The horizontal red and blue lines indicate the genome-wide significant (*P* = 5.12 × 10^–9^) and suggestive (*P* = 1.02 × 10^–7^) thresholds, respectively. **C** Venn diagram of 3 overlapping SNPs (rs317934171, rs14200392 and rs731580755) based on the GLM and eGWAS. GLM: general linear model, eGWAS: expression genome-wide association study. **D** and **E** Differences in the EW and BMW between different genotypes of rs317934171. ns: *P* > 0.05, **: *P* < 0.01, BMW: breast muscle weight, EW: eviscerated weight. **F** Differences in the *SERPINB6* expression level between different genotypes of rs317934171. ns: *P* > 0.05, **: *P* < 0.01, ***: *P* < 0.001, ****: *P* < 0.0001

To identify the key variants affecting the expression of *SERPINB6*, we performed an eGWAS to detect the molecular markers. A ~11.59 kb region located on chicken (*Gallus gallus*) chromosome 2 (31.67–43.26 kb) showed a significant peak for *SERPINB6* expression (Fig. 4A). Within this region, rs317934171 and rs313806168 were identified as being above the genome-wide significance threshold (*P* = 5.12 × 10^–9^). No population stratification was found based on the calculated genomic inflation factor (1.019) in the Q‒Q plot (Fig. 4B). A total of 8 SNPs over the suggestive threshold were observed. The lead SNP (rs317934171) had the highest allele frequency, and was located in the functional region which was the 3′UTR of *SERPINB6* (Table 3). To further evaluate the relationships between genotype and phenotype (4 carcass traits), we used the GLM for association analysis. Fifteen SNPs were significantly associated with the 4 carcass traits in 357 samples (Additional file 14: Table S7). Among them, rs317934171, rs14200392 and rs731580755 were overlapping SNPs (Fig. 4C).

**Table 3:** Information on SNPs significantly associated with the expression of *SERPINB6*

Chr^1^	Position	Variant ID	Alleles	*P* value	MAF^2^	Annotation
2	67631671	rs317934171	C/T	6.60E-09	0.469	3′UTR variant
2	67642960	rs313806168	G/A	6.89E-09	0.345	Upstream gene variant
2	67641980	rs313663866	G/A	1.61E-08	0.322	Upstream gene variant
2	67643255	rs14200402	A/G	4.08E-08	0.350	Upstream gene variant
2	67637887	rs14200392	G/C	4.30E-08	0.439	Intron variant
2	67637347	rs317512789	A/G	5.92E-08	0.381	Intron variant
2	67639396	rs731580755	C/T	6.08E-08	0.445	Intron variant
2	67643258	rs14200403	C/T	8.75E-08	0.348	Upstream gene variant

^1^*Chr* Chromosome

^2^*MAF* Minor allele frequency

### rs317934171 is a variant of *SERPINB6*

In Tiannong partridge chickens, individuals with the CC genotype had significantly higher eviscerated weight and breast muscle weight than did those with the TT genotype (Fig. 4D and E). Moreover, *SERPINB6* expression showed the same trend that *SERPINB6* expression was greater in the CC genotype than those in the TT genotype (Fig. 4F). Accordingly, rs317934171 was considered a key SNP associated with *SERPINB6* expression based on the results of eGWAS and association analysis. In addition, we found that the CC genotype increased the promoter activity of *SERPINB6* using dual-luciferase assays (Additional file 15: Fig. S8). Considering that the SNP is located in the 3′UTR, it was surprising that the transcription factor *Adf-1* was only associated with the CC genotype of rs317934171, as determined by PROMO software (Additional file 16: Fig. S9). Therefore, we believe that the binding of the *Adf-1* transcription factor to the key variant rs317934171 may be the key to improving *SERPINB6* expression and meat production.

### gga-miR-1615 binds to *SERPINB6*

**Fig. 5:**
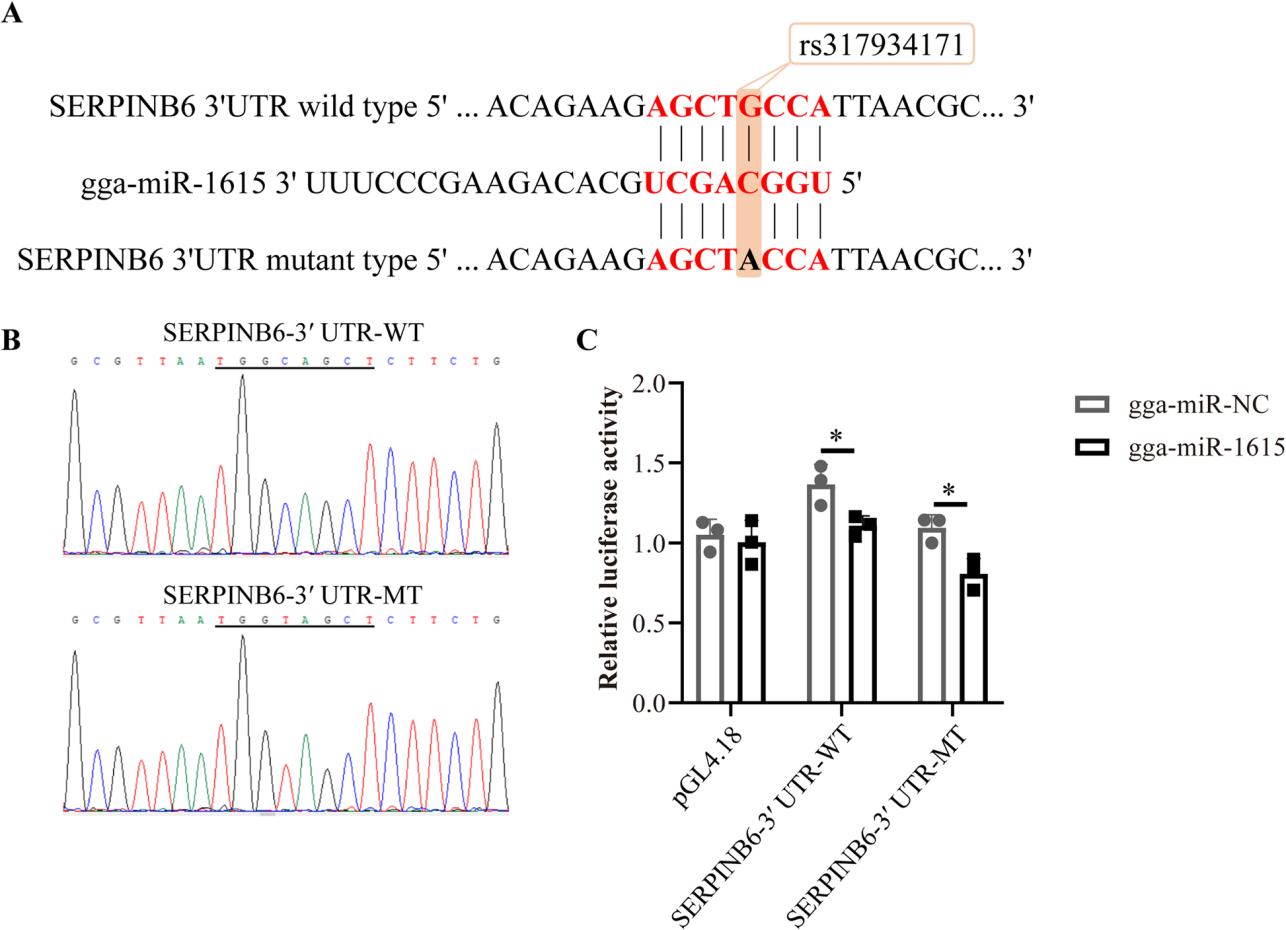
gga-miR-1615 directly targets *SERPINB6*. **A** Complementary pairing of gga-miR-1615 with the wild type or mutant 3′UTR of the target gene, *SERPINB6*. **B** Validation of the wild type and mutant type by Sanger sequencing. WT indicates wild type and MT indicates mutant type. **C** HEK293 cells were co-transfected with the wild type or mutant *SERPINB6* 3′UTR dual-luciferase vector and the miR-1615 mimic or mimic NC. The data represent the mean ± 3 standard deviations from 3 biological replicates. *: *P* < 0.05

Based on the identified key variants located in the 3′UTR, we asked whether miRNAs binding blocks translation of the *SERPINB6* gene. Using the miRDB software, we predicted that gga-miR-1615 would act on the 3′UTR of *SERPINB6* according to the principle of complementary base pairing. Coincidentally, the seed sequence “AGCTGCCA” contains rs317934171 (Fig. 5A). We confirmed the authenticity of rs317934171 by Sanger sequencing (Fig. 5B). To further verify the relationship between gga-miR-1615 and *SERPINB6*, we constructed a dual-luciferase reporter vector. After co-transfecting the dual luciferase reporter plasmids (pGL4.18 and WT/MT) and gga-miR-NC/gga-miR-1615, we found that gga-miR-1615 significantly decreased the dual luciferase activity compared with that obtained with gga-miR-NC (Fig. 5C). However, the expression of *SERPINB6* did not change significantly after transfection with 4 siRNAs, namely, mimic-NC, miR-1615-mimic, inhibitor-NC and miR-1615-inhibitor, in DF1 cells and CPMs (Additional file 17: Fig. S10). Therefore, our results indicate that gga-miR-1615 affects the translation of *SERPINB6* and that other miRNAs may also play a role in this process.

## Discussion

**Fig. 6:**
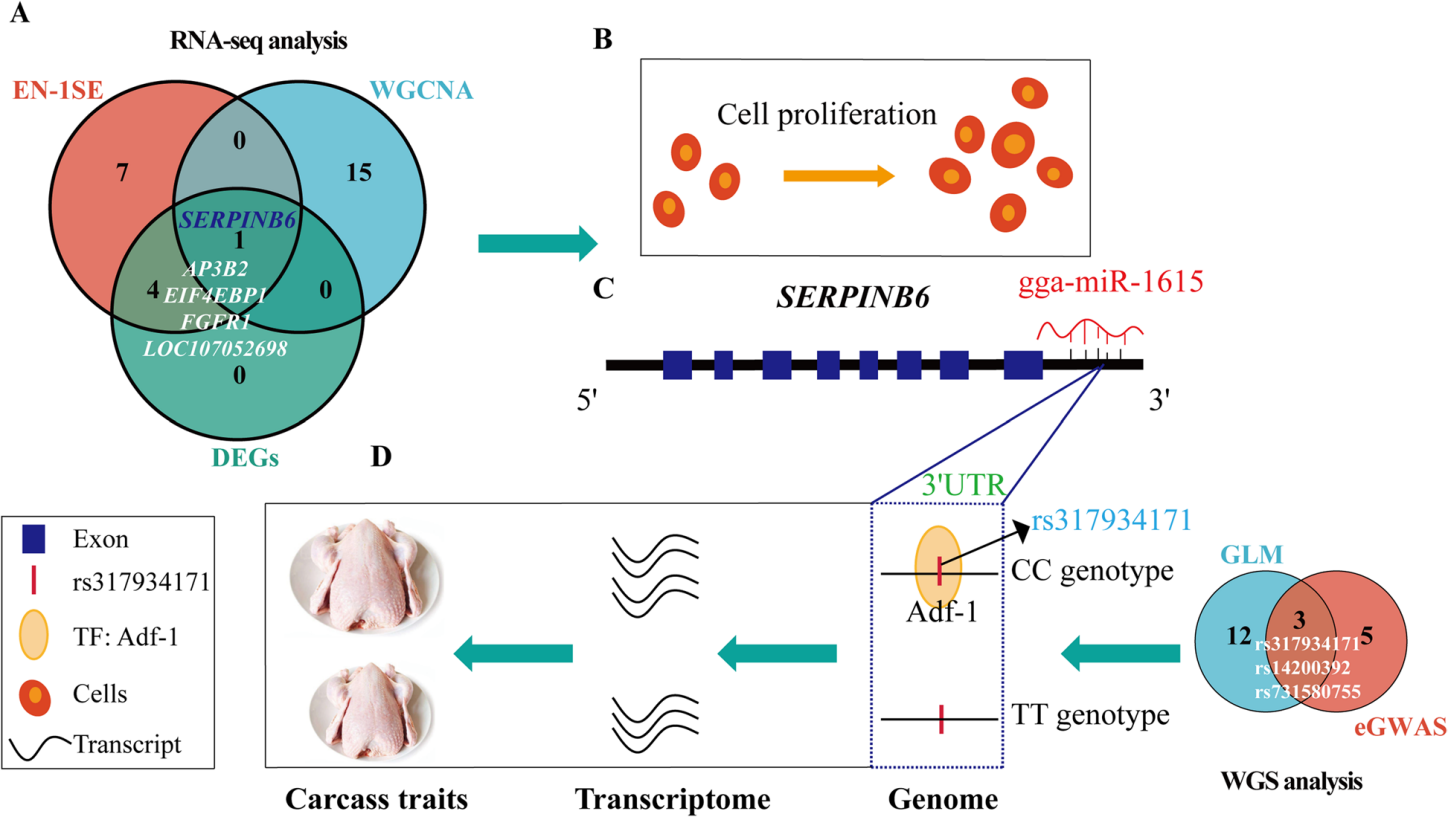
Map of a novel functional gene, *SERPINB6*, for carcass traits and a key variant, rs317934171, located in the 3′UTR of the gene. **A** Venn diagram of 1 overlapping gene (*SERPINB6*) associated with the EW based on RNA-seq data. EN-1SE with parameters determined based on the one standard error rule, WGCNA: weighted gene co-expression network analysis, DEGs: differentially expressed genes. **B** *SERPINB6* promotes DF1 cell and CPMs proliferation. **C** Interaction between gga-miR-1615 and *SERPINB6*. **D** A key molecular marker (rs317934171) in the 3′UTR has significant effects on increasing *SERPINB6* expression, EW and BMW, as revealed by GLM and eGWAS. And *Adf-1* only binds to the CC genotype of rs317934171. TF: transcription factor, GLM: general linear model, WGS: whole-genome sequencing

The Tiannong partridge chicken is a commercially available 3-way cross chicken produced from 3 pure lines of Qingyuan partridge chicken and Guangxi partridge chicken; this chicken is an important indigenous Chinese slow-growing yellow-feathered chicken and is well known for its superior meat quality [21]. Few key candidate genes for carcass traits have been identified, and the molecular mechanisms regulating carcass traits are unclear. Here, based on large-scale RNA-seq and WGS, we identified key genes and molecular markers associated with carcass traits, particularly EW (Fig. 6).

### Advantages of the EN-1SE model for screening candidate genes

At present, one or two analyses are generally used to identify candidate genes by RNA-seq [33]. However, the main advantage of this study is that candidate genes were screened by 3 methods, namely EN-1SE model, WGCNA and differential expression analysis. First, our previous research showed the following: LMM, LASSO-1SE and EN-1SE had the lowest type I error rates, which were exactly zero; EN-Min minimized cross-validated mean squared prediction error [34] with an additional significance test; and LASSO-Min showed type I error rates of less than 0.02. EN-Min without significance testing and RR-1SE had high type I error rates, although these were lower than those of RR-Min. Taken together, the results of the simulation study demonstrated that EN-1SE without significance testing was the optimal method for analyzing associations of expression levels with traits in terms of both the type I error rate and the detection power [21].

Based on the advantages of EN-1SE, 12 genes were found to be significantly associated with 4 carcass traits. Some genes were reported to be associated with carcass traits or muscle development. For example, *AMY1A* polymorphisms were found to be associated with growth, carcass traits and feed intake efficiency in chickens, including daily gain, average daily feed intake, leg muscle weight and abdominal fat (*P* < 0.05) [35]. *CEBPG* is a transcription factor that binds to the promoter and enhancer regions of target genes [36], and *ATF5* in combination with *CEBPG* only reduces the size of glycolytic fibers and tends to increase the proportion of oxidative fibers [37]. *EIF4EBP1*, which is resistant to mTORC1-mediated inhibition, preserves muscle function by increasing mitochondrial activities [38]. *FGFR1* can bind to LBP1C-2 to activate satellite cells and promote satellite cell self-renewal through upregulation of *SPAY1* [39], and may play a role regulating postnatal skeletal muscle maintenance [40].

### Characteristics of WGCNA screening for hub genes

The second method, WGCNA, is a systematic analysis method used to describe gene association patterns [41, 42]. This method can divide thousands of genes into multiple modules according to the gene expression patterns and identify hub genes in the modules and is instrumental for improving the accuracy and reducing the number of calculations. Among the 26 hub genes (e.g., *CREBBP*, *ZNF335*, and *SERPINB6*) we identified, the regulatory mechanism for genes associated with carcass traits is largely unknown. According to previous reports, the *CREBBP* gene is ubiquitously expressed and is involved in the transcriptional co-activation of many different transcription factors [43]. *ZNF335* is essential for self-renewal of neural progenitors, neurogenesis, and neuronal differentiation [44].

### Function of *SERPINB6*

We found that *SERPINB6* was the only overlapping gene between the 3 analyses. *SERPINB6* encodes a member of the serine proteinase inhibitor (serpin) superfamily and the ovalbumin serpin subfamily, which are involved in cell migration, cell differentiation and apoptosis [45]. *SERPINB6* is abundantly expressed by mast cells in all organs and by the human mast cell line HMC-1 [46]. Further studies have shown that *SERPINB6* can regulate the activity of endogenous trypsin in the cytoplasm and is significantly negatively correlated with deer meat shear force and intramuscular fat, with correlation coefficients of −0.734 and −0.816, respectively [47]. Recent research has shown that *SERPINB6* is a target protein of dutomycin. For the first time, dutomycin was shown to directly bind to *SERPINB6*, activate an intracellular serine protease and induce autophagy [48].

### *SERPINB6* promotes cell proliferation

To date, *SERPINB6* has not been reported to be significantly associated with carcass traits, and the molecular mechanism by which *SERPINB6* affects carcass traits is also unknown. We then selected DF1 cells and CPMs to verify the new function of *SERPINB6* in chickens. Here, we constructed an experimental model for the proliferation of DF1 cells and CPMs after *SERPINB6* knockdown or overexpression. Through a series of in vitro experiments, CCK8 and EdU assays indicated that *SERPINB6* overexpression promoted the proliferation of DF1 cells and CPMs, and *SERPINB6* knockdown inhibited the proliferation of DF1 cells and CPMs.

### *SERPINB6* overexpression results in the formation of a complex regulatory network

Moreover, thousands of DEGs (e.g., *MYC* [49], *ACTA2*, and *MYH10* [50]) were enriched mainly after *SERPINB6* overexpression, mainly in tight junction, ECM-receptor interaction, focal adhesion, and MAPK signaling pathway and so on. Tight junction proteins contribute to the control of cell proliferation [51]. Transcriptome analysis revealed that the ECM-receptor interaction pathway is associated with meat quality in broiler chickens [52] and can directly or indirectly control cellular activities such as adhesion, migration, differentiation, proliferation, and apoptosis. The focal adhesion pathway plays an essential role in important biological processes such as cell motility, cell proliferation, cell differentiation and gene expression regulation [53, 54]. Therefore, *SERPINB6* promotes cell proliferation through a complex regulatory network.

### eGWAS identifies associations between genetic variants and *SERPINB6* expression

We identified associations between genetic variation and *SERPINB6* expression through eGWAS that is to screen for key SNPs and laying the foundation for further elaboration of the mechanism affecting gene expression levels [55]. Our results indicated that eight variants associated with *SERPINB6* (67,630,787–67,640,291) were located in this region (67,631,671–67,643,258), and these SNPs were the *cis*-eQTLs.

We finally identified 3 molecular markers (rs317934171, rs14200392 and rs731580755) by eGWAS and association analysis, and rs317934171 happened to be located in the 3′UTR of *SERPINB6*. With the SNP serving as a molecular marker, we found that the SNP increased *SERPINB6* expression. The EW and BMW of the CC genotype were significantly higher than those of the TT genotype, with increases of 4.60% and 5.12%, respectively. Notably, the proportion of *SERPINB6* in the CC genotype was 26.28% higher than that in the TT genotype. At the same time, the wild type (CC genotype) significantly enhanced luciferase activity. Therefore, the CC genotype has a greater effect on rs317934171. The CC genotype is predicted to bind to the *Adf-1* transcription factor, which can enhance the promoter activity of *SERPINB6* and promote its expression. *Adf-1* has also been reported to be a key modulator of the dynamic organization of the actin cytoskeleton [56]. Further validation of the interaction between *Adf-1* and rs317934171 or *SERPINB6* is needed.

### gga-miR-1615 binds to *SERPINB6*

In general, the 3′UTR of genes often binds to miRNAs. For example, miR-21-5p regulates skeletal muscle satellite cell proliferation and differentiation by targeting *KLF3* in the 3′UTR in chickens [57]. Cai et al. [58], confirmed that miR-16-5p, which significantly decreased luciferase activity, targeted *SESN1* to regulate the p53 signaling pathway, affecting myoblast proliferation and apoptosis. We found that gga-miR-1615 significantly decreased double luciferase activity, which is consistent with previous research reports [57, 58].

Moreover, transfection of the gga-miR-1615 mimic and gga-miR-1615 inhibitor did not significantly change the *SERPINB6* expression levels in DF1 cells, but the *SERPINB6* expression levels were significantly reduced after transfection of the gga-miR-1615 inhibitor in CPMs. Multiple miRNAs may silence gene expression to further induce phenotypic changes, or both gga-miR-1615 and *Adf-1* may change *SERPINB6* expression. More studies on this article will be performed in the future.

## Conclusion

Overall, we identified *SERPINB6* as a novel functional gene that is significantly associated with carcass traits by association analysis, including the EN-1SE model, WGCNA and differential expression analysis, based on RNA-seq data. Furthermore, our results suggested that rs317934171 of the CC genotype, as identified through eGWAS and association analysis, significantly increases *SERPINB6* expression levels, and that *SERPINB6* promoted cell proliferation in DF1 cells and CPMs. Furthermore, a direct target region relationship was found between gga-miR-1615 and *SERPINB6*. We explored the biological function and preliminary regulatory mechanism of *SERPINB6* and identified its molecular markers.

## Supplementary Information

**Additional file 1: Fig. S1.** Low group and high group for 4 carcass traits.

**Additional file 2: Table S1.** RNA oligonucleotides used in this study.

**Additional file 3: Table S2.** Primer pairs for RT‒PCR.

**Additional file 4: Fig. S2.** Spearman correlation analysis of the 4 carcass traits (*n* = 381).

**Additional file 5: Table S3.** Genes with non-zero effects on each carcass trait for the EN-1SE model.

**Additional file 6: Fig. S3.** Venn diagram of 12 overlapping genes with non-zero effects on carcass traits based on the EN-1SE model.

**Additional file 7: Fig. S4.** Weighted gene co-expression network analysis.

**Additional file 8: Table S4.** Hub genes for target modules.

**Additional file 9: Fig. S5.** Volcano map of 4 carcass traits.

**Additional file 10: Table S5.** DEGs associated with carcass traits.

**Additional file 11: Fig. S6.** Transfection efficiency of *SERPINB6* in DF1 cells and CPMs.

**Additional file 12: Table S6.** Thirty-eight KEGG pathways and 456 GO terms were enriched.

**Additional file 13: Fig. S7.** Protein–protein interaction network of DEGs in DF1 cells.

**Additional file 14: Table S7.** Comparison of the phenotypes of different genotypes with significant association sites of *SERPINB6*.

**Additional file 15: Fig. S8.** rs317934171 enhances promoter activity.

**Additional file 16: Fig. S9.** Prediction of transcription factors before and after mutation of rs317934171.

**Additional file 17: Fig. S10.** mRNA expression levels of *SERPINB6* after transfection of mimic-NC, miR-1615-mimic, inhibitor-NC and miR-1615-inhibitor in DF1 cells and CPMs.

## Data Availability

The datasets analyzed during the current study are available from the corresponding author upon reasonable request, and we’ve uploaded them to Genome Sequence Archive (https://ngdc.cncb.ac.cn/gsa) under the BioProject no. CRA014989 and CRA015111.

## Abbreviations

BMW: Breast muscle weight
CCK8: Cell Counting Kit-8
CV: Coefficient of variation
DEGs: Differentially expressed genes
DF1: Chicken embryonic fibroblasts line
DW: Dressed weight
EdU: 5-Ethynyl-2-deoxyuridine
EN-1SE: Elastic net-1 standard error
eGWAS: Expression genome-wide association study
FBS: Fetal bovine serum
GLM: General linear model
GO: Gene Ontology
GS: Gene significance
GWAS: Genome-wide association study
KEGG: Kyoto Encyclopedia of Genes and Genomes
LW: Live weight
MAF: Minor allele frequency
MM: Module membership
MT: Mutant type
RNA-seq: Transcriptome sequencing
RT‒PCR: Real time-polymerase chain reaction
SD: Standard deviation
siRNAs: Small interfering RNAs
SNP: Single-nucleotide polymorphism
WGCNA: Weighted gene co-expression network analysis
WGS: Whole-genome sequencing
WT: Wild type
