# Supplementary figures and images for: Large-scale transcriptomic and genomic analyses reveal a novel functional gene SERPINB6 for chicken carcass traits
Source: J Anim Sci Biotechnol. 2024 May 11;15:70. doi: 10.1186/s40104-024-01026-3 (PMC11571647; doi:10.1186/s40104-024-01026-3)

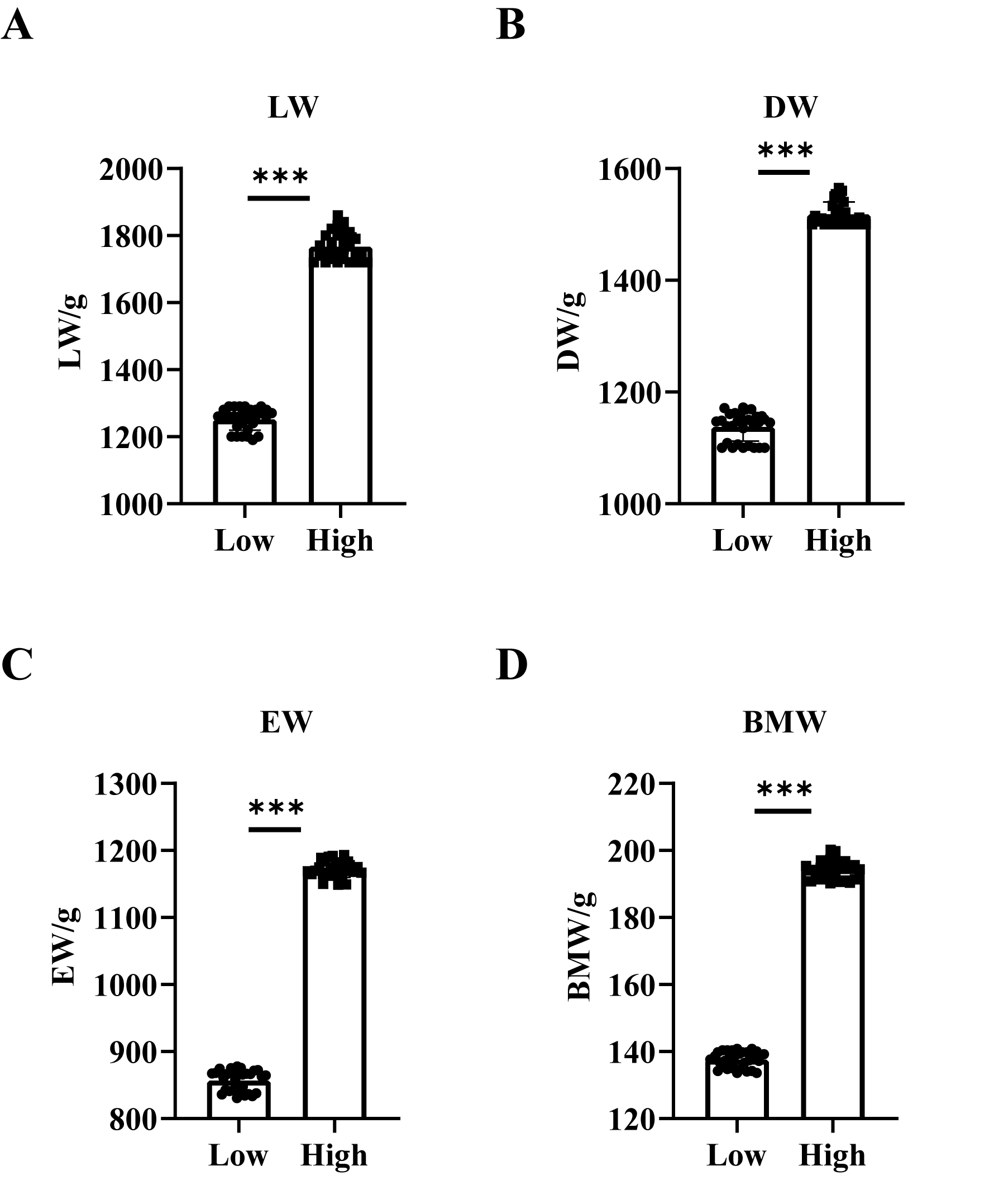

Supplement: Supplementary file 1 — Additional file 1: Fig. S1. Low group and high group for 4 carcass traits. [file 40104_2024_1026_MOESM1_ESM.tif]

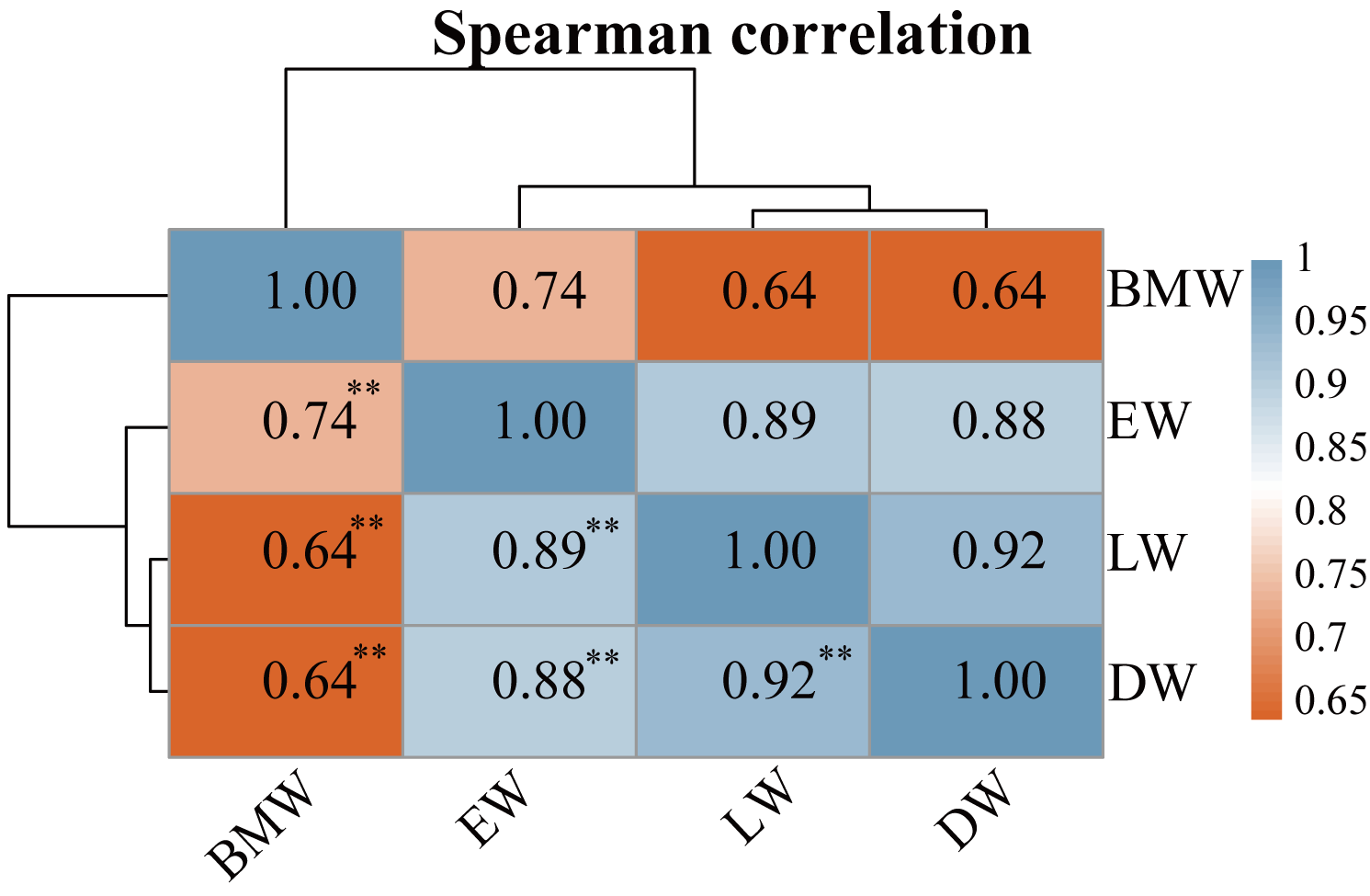

Supplement: Supplementary file 4 — Additional file 4: Fig. S2. Spearman correlation analysis of the 4 carcass traits (n = 381). [file 40104_2024_1026_MOESM4_ESM.tif]

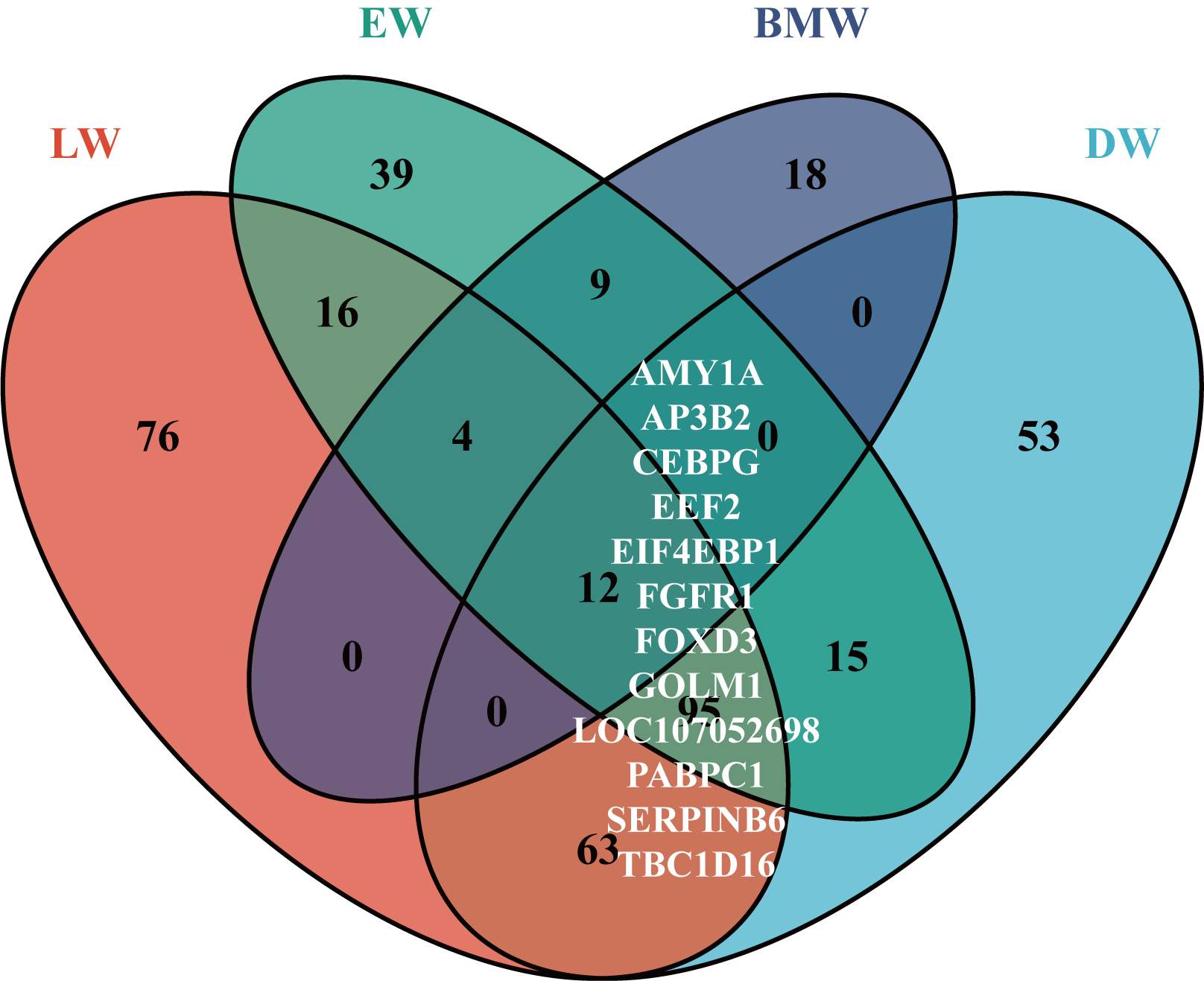

Supplement: Supplementary file 6 — Additional file 6: Fig. S3. Venn diagram of 12 overlapping genes with non-zero effects on carcass traits based on the EN-1SE model. [file 40104_2024_1026_MOESM6_ESM.tif]

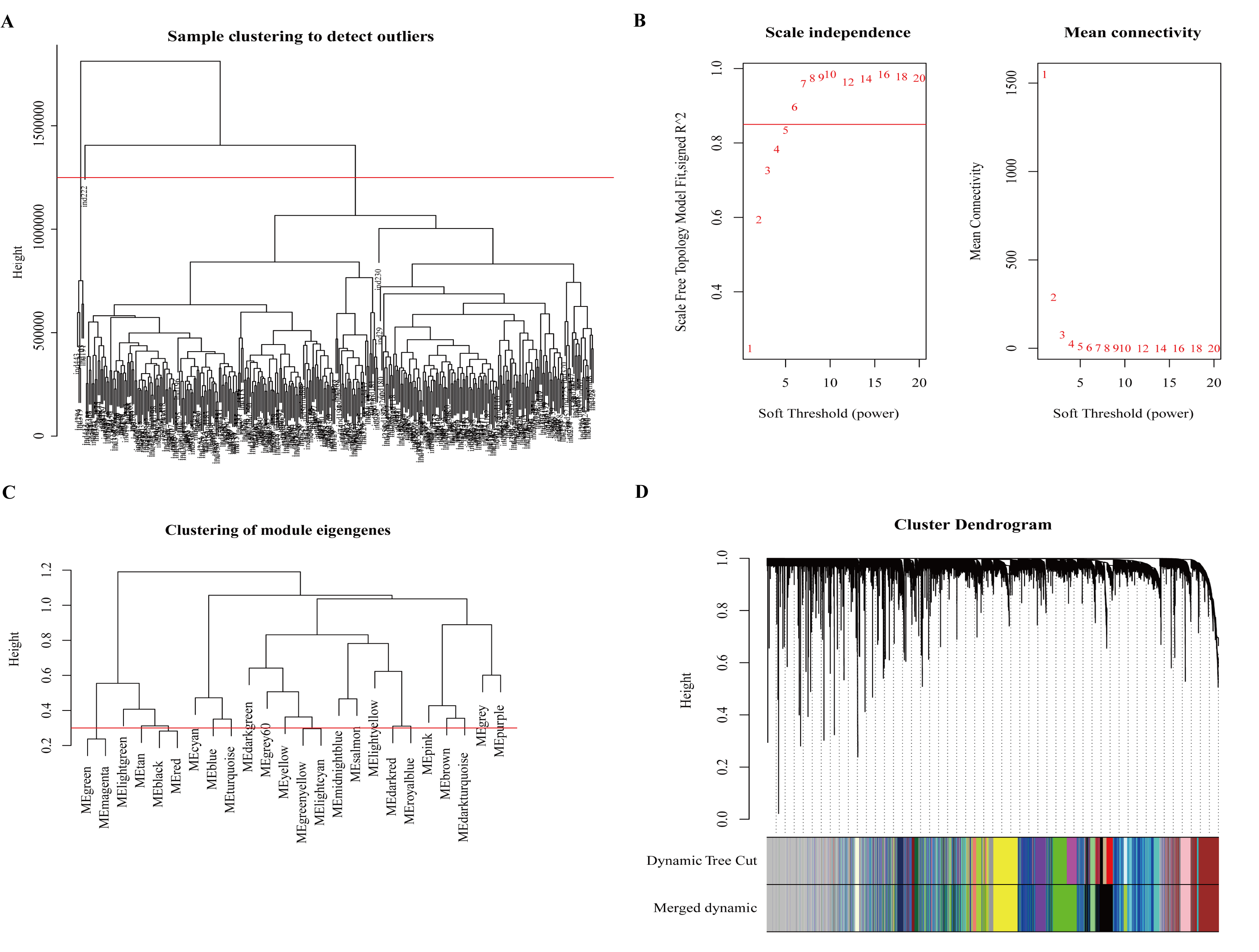

Supplement: Supplementary file 7 — Additional file 7: Fig. S4. Weighted gene co-expression network analysis. [file 40104_2024_1026_MOESM7_ESM.tif]

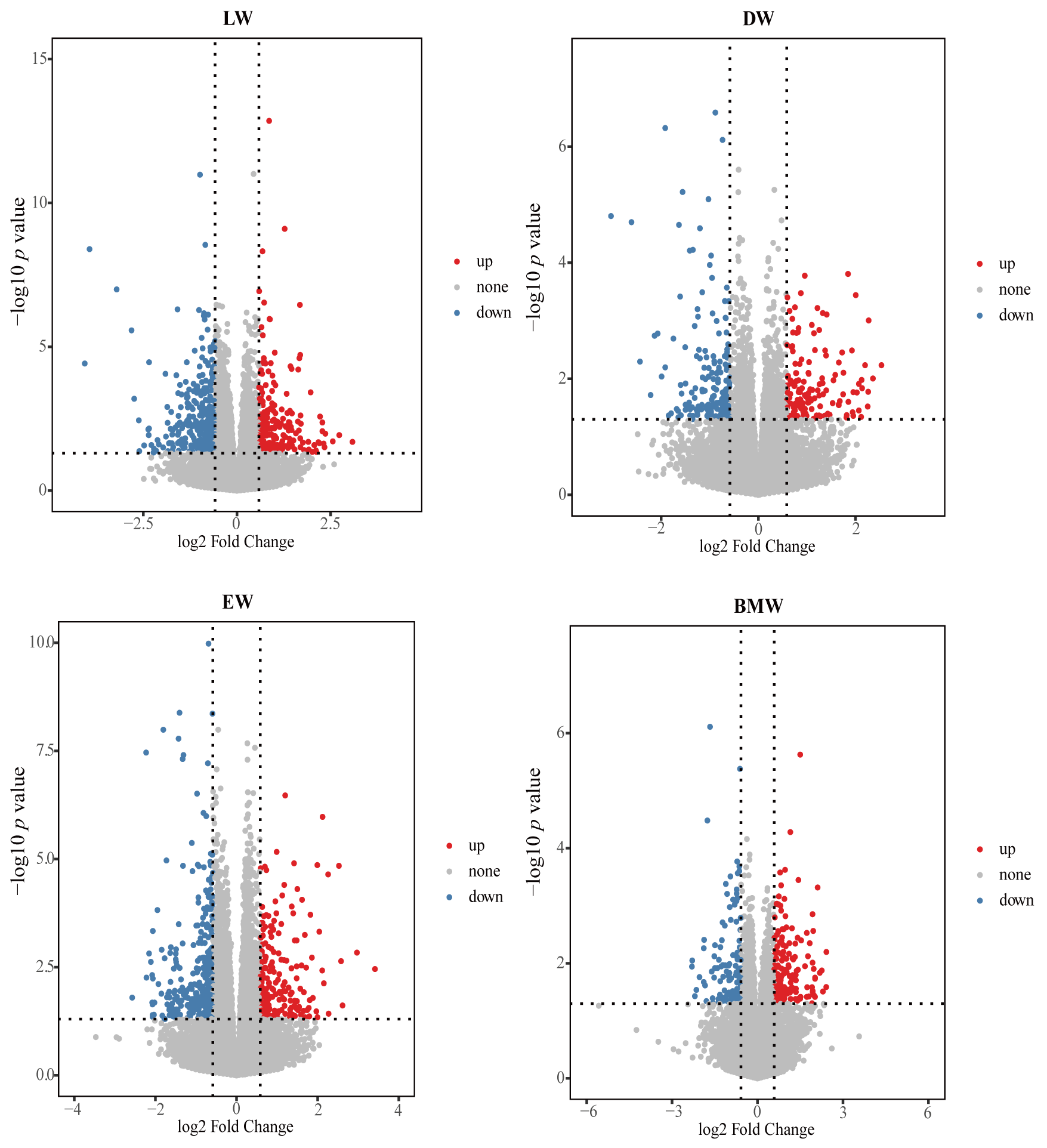

Supplement: Supplementary file 9 — Additional file 9: Fig. S5. Volcano map of 4 carcass traits. [file 40104_2024_1026_MOESM9_ESM.tif]

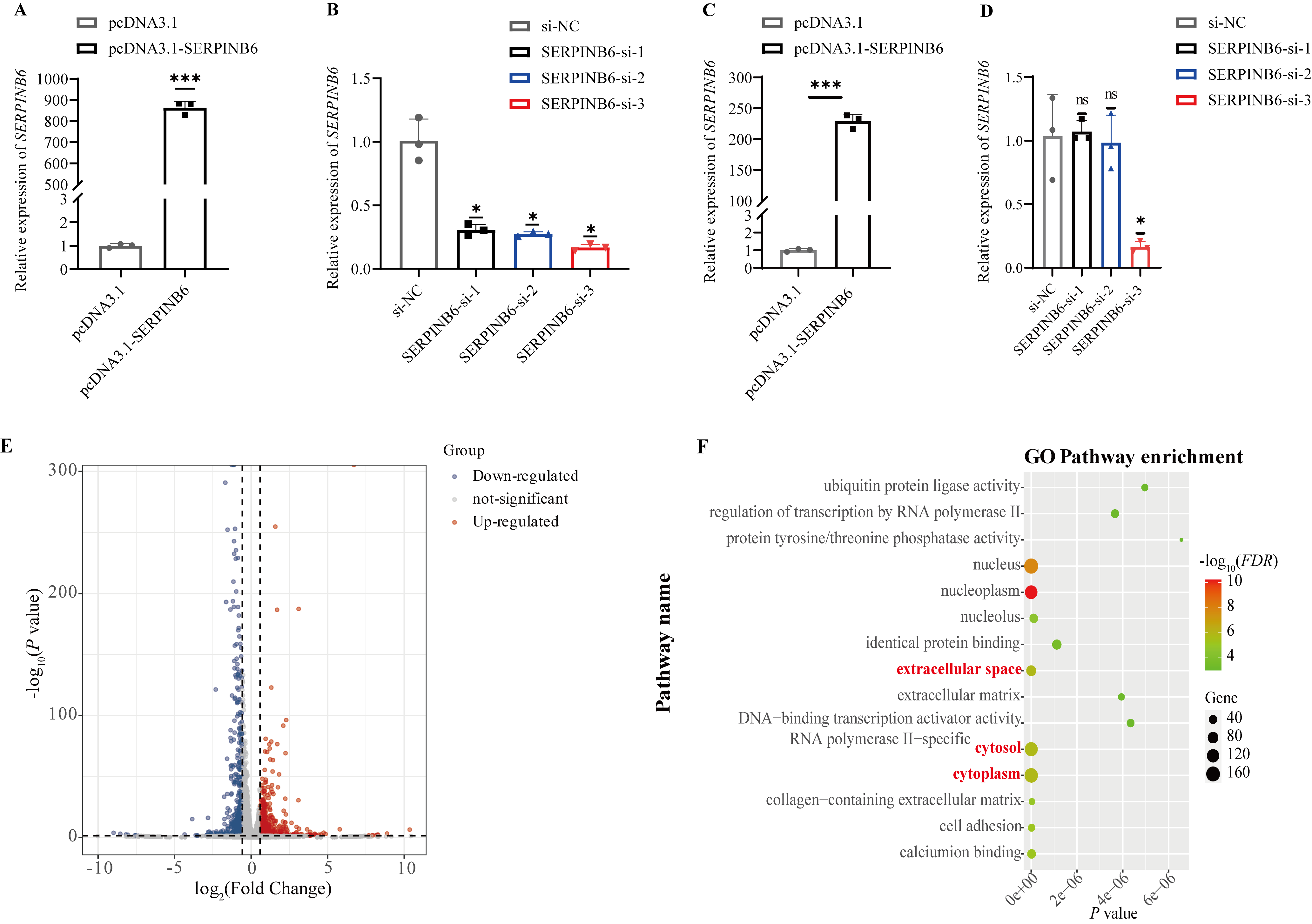

Supplement: Supplementary file 11 — Additional file 11: Fig. S6. Transfection efficiency of SERPINB6 in DF1 cells and CPMs. [file 40104_2024_1026_MOESM11_ESM.tif]

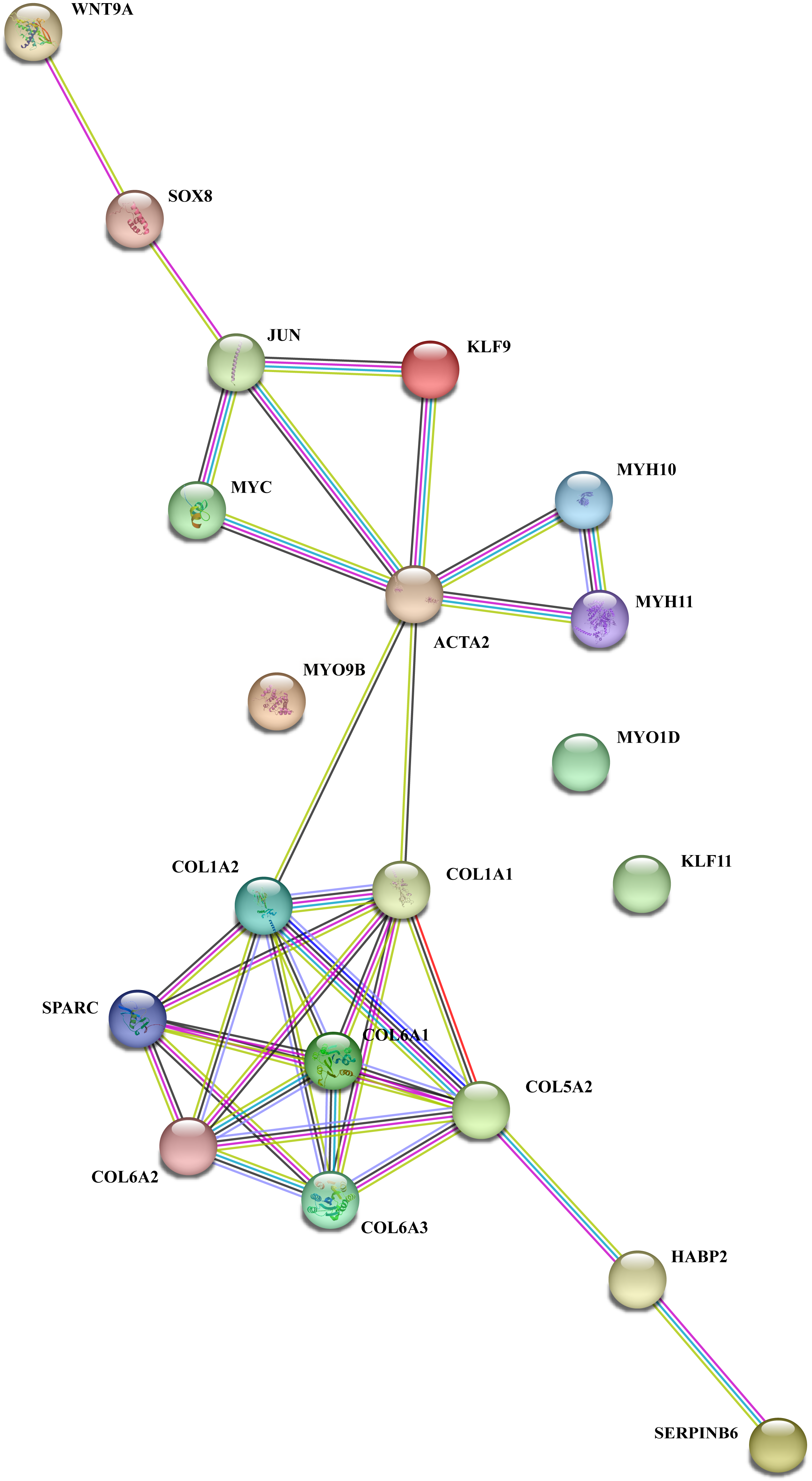

Supplement: Supplementary file 13 — Additional file 13: Fig. S7. Protein–protein interaction network of DEGs in DF1 cells. [file 40104_2024_1026_MOESM13_ESM.tif]

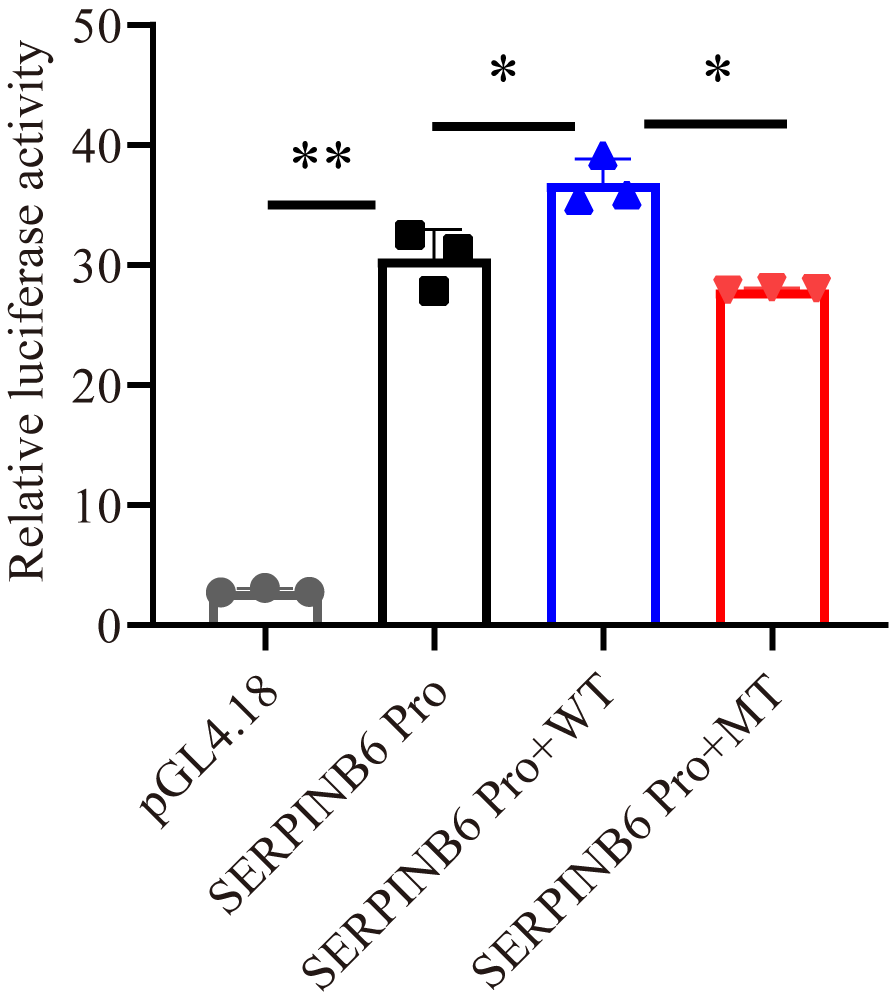

Supplement: Supplementary file 15 — Additional file 15: Fig. S8. rs317934171 enhances promoter activity. [file 40104_2024_1026_MOESM15_ESM.tif]

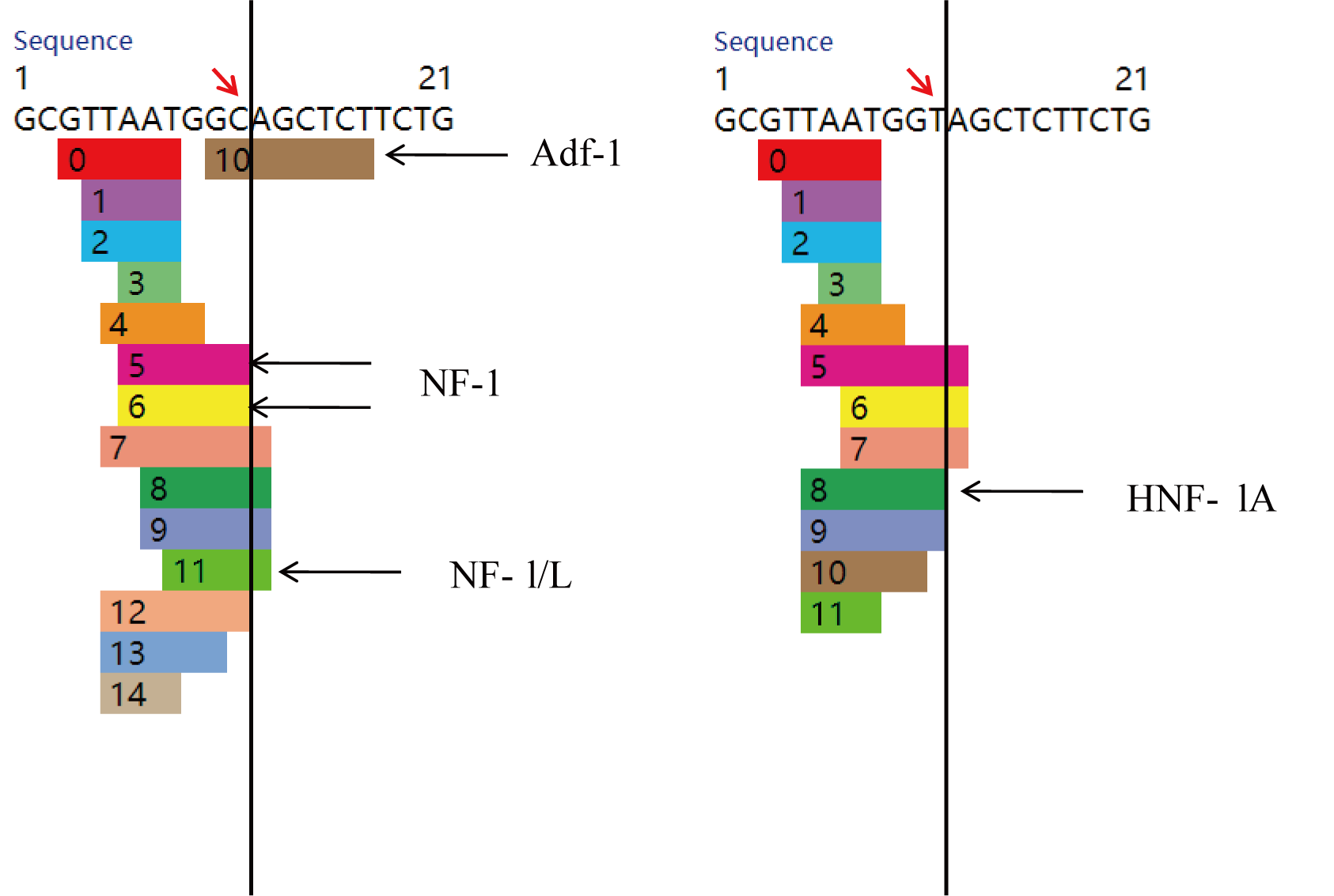

Supplement: Supplementary file 16 — Additional file 16: Fig. S9. Prediction of transcription factors before and after mutation of rs317934171. [file 40104_2024_1026_MOESM16_ESM.tif]

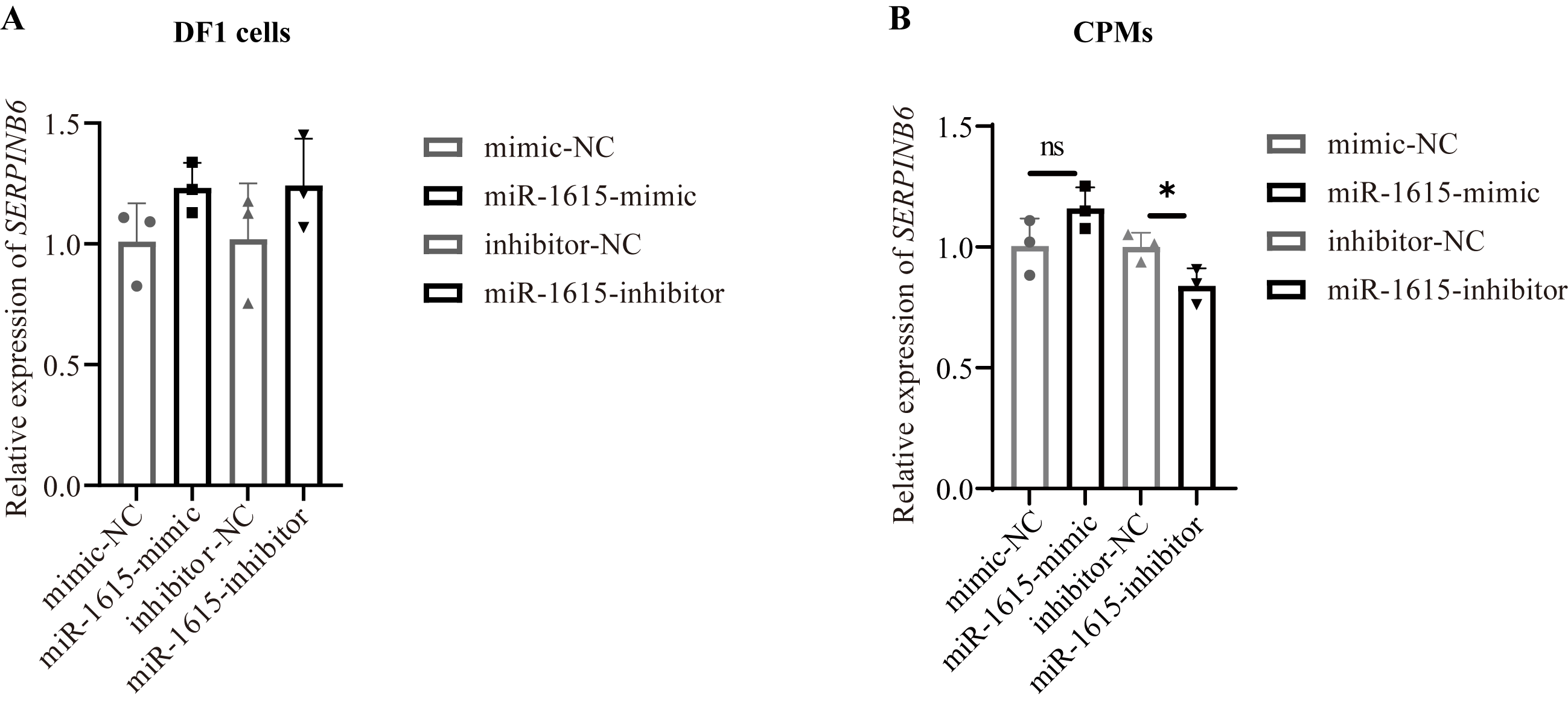

Supplement: Supplementary file 17 — Additional file 17: Fig. S10. mRNA expression levels of SERPINB6 after transfection of mimic-NC, miR-1615-mimic, inhibitor-NC and miR-1615-inhibitor in DF1 cells and CPMs. [file 40104_2024_1026_MOESM17_ESM.tif]
